# Long runs of homozygosity are associated with Alzheimer’s disease

**DOI:** 10.1038/s41398-020-01145-1

**Published:** 2021-02-24

**Authors:** Sonia Moreno-Grau, Maria Victoria Fernández, Itziar de Rojas, Pablo Garcia-González, Isabel Hernández, Fabiana Farias, John P. Budde, Inés Quintela, Laura Madrid, Antonio González-Pérez, Laura Montrreal, Emilio Alarcón-Martín, Montserrat Alegret, Olalla Maroñas, Juan Antonio Pineda, Juan Macías, The GR@ACE study group, C. Abdelnour, N. Aguilera, E. Alarcón-Martín, M. Alegret, A. Benaque, M. Boada, M. Buendía, P. Cañabate, A. Carracedo, A. Corbatón, I. de Rojas, S. Diego, A. Espinosa, A. Gailhajenet, P. García González, S. Gil, M. Guitart, A. González Pérez, I. Hernández, M. Ibarria, A. Lafuente, J. Macías, O. Maroñas, E. Martín, M. T. Martínez, M. Marquié, A. Mauleón, G. Monté-Rubio, L. Montrreal, S. Moreno-Grau, M. Moreno, A. Orellana, G. Ortega, A. Pancho, E. Pelejà, A. Pérez-Cordon, J. A. Pineda, S. Preckler, I. Quintela, L. M. Real, O. Rodríguez-Gómez, M. Rosende-Roca, A. Ruiz, S. Ruiz, M. E. Sáez, A. Sanabria, M. A. Santos-Santos, M. Serrano-Ríos, O. Sotolongo-Grau, L. Tárraga, S. Valero, L. Vargas, DEGESCO consortium, A. D. Adarmes-Gómez, E. Alarcón-Martín, I. Álvarez, V. Álvarez, G. Amer-Ferrer, M. Antequera, C. Antúnez, M. Baquero, M. Bernal, R. Blesa, M. Boada, D. Buiza-Rueda, M. J. Bullido, J. A. Burguera, M. Calero, F. Carrillo, M. Carrión-Claro, M. J. Casajeros, J. Clarimón, J. M. Cruz-Gamero, M. M. de Pancorbo, I. de Rojas, T. del Ser, M. Diez-Fairen, J. Fortea, E. Franco, A. Frank-García, J. M. García-Alberca, S. García Madrona, G. Garcia-Ribas, P. Gómez-Garre, I. Hernández, S. Hevilla, S. Jesús, M. A. Labrador Espinosa, C. Lage, A. Legaz, A. Lleó, A. López de Munáin, S. López-García, D. Macías, S. Manzanares, M. Marín, J. Marín-Muñoz, T. Marín, M. Marquié, A. Martín-Montes, B. Martínez, C. Martínez, V. Martínez, P. Martínez-Lage Álvarez, M. Medina, M. Mendioroz Iriarte, M. Menéndez-González, P. Mir, J. L. Molinuevo, L. Montrreal, S. Moreno-Grau, A. Orellana, A. B. Pastor, P. Pastor, J. Pérez-Tur, T. Periñán-Tocino, G. Piñol-Ripoll, A. Rábano, D. Real de Asúa, S. Rodrigo, E. Rodríguez-Rodríguez, J. L. Royo, A. Ruiz, R. Sanchez del Valle Díaz, P. Sánchez-Juan, I. Sastre, O. Sotolongo-Grau, L. Tárraga, S. Valero, M. P. Vicente, L. Vivancos, Marta Marquié, Sergi Valero, Alba Benaque, Jordi Clarimón, Maria Jesus Bullido, Guillermo García-Ribas, Pau Pástor, Pascual Sánchez-Juan, Victoria Álvarez, Gerard Piñol-Ripoll, Jose María García-Alberca, José Luis Royo, Emilio Franco-Macías, Pablo Mir, Miguel Calero, Miguel Medina, Alberto Rábano, Jesús Ávila, Carmen Antúnez, Luis Miguel Real, Adelina Orellana, Ángel Carracedo, María Eugenia Sáez, Lluís Tárraga, Mercè Boada, Carlos Cruchaga, Agustín Ruiz, for the Alzheimer’s Disease Neuroimaging Initiative

**Affiliations:** 1 Research Center and Memory clinic Fundació ACE. Institut Català de Neurociències Aplicades, Universitat Internacional de Catalunya, Barcelona, Spain; 2 CIBERNED, Center for Networked Biomedical Research on Neurodegenerative Diseases, Carlos III Institute of Health, Madrid, Spain; 3 Department of Psychiatry, Washington University School of Medicine, St. Louis, MO United States of America; 4 Hope Center for Neurological Disorders, Washington University School of Medicine, St. Louis, MO United States of America; 5 Grupo de Medicina Xenómica, Centro Nacional de Genotipado (CEGEN-PRB3-ISCIII), Universidade de Santiago de Compostela, Santiago de Compostela, Spain; 6 CAEBI. Centro Andaluz de Estudios Bioinformáticos, Sevilla, Spain; 7 Unidad Clínica de Enfermedades Infecciosas y Microbiología. Hospital Universitario de Valme, Sevilla, Spain; 8 Memory Unit, Neurology Department and Sant Pau Biomedical Research Institute, Hospital de la Santa Creu i Sant Pau, Universitat Autònoma de Barcelona, Barcelona, Spain; 9 Centro de Biología Molecular Severo Ochoa (C.S.I.C.-U.A.M.), Universidad Autónoma de Madrid, Madrid, Spain; 10 Instituto de Investigación Sanitaria “Hospital la Paz” (IdIPaz), Madrid, Spain; 11 Hospital Universitario Ramón y Cajal, Madrid, Spain; 12 Fundació per la Recerca Biomèdica i Social Mútua Terrassa, and Memory Disorders Unit, Department of Neurology, Hospital Universitari Mútua de Terrassa, University of Barcelona School of Medicine, Terrassa, Barcelona, Spain; 13 Neurology Service “Marqués de Valdecilla” University Hospital (University of Cantabria and IDIVAL), Santander, Spain; 14 Laboratorio de Genética Hospital Universitario Central de Asturias, Oviedo, Spain; 15 Instituto de Investigación Biosanitaria del Principado de Asturias (ISPA), Oviedo, Spain; 16 Unitat Trastorns Cognitius, Hospital Universitari Santa Maria de Lleida, Institut de Recerca Biomédica de Lleida (IRBLLeida), Lleida, Spain; 17 Alzheimer Research Center & Memory Clinic, Andalusian Institute for Neuroscience, Málaga, Spain; 18 Dep. of Surgery, Biochemistry and Molecular Biology, School of Medicine, University of Málaga, Málaga, Spain; 19 Unidad de Demencias, Servicio de Neurología y Neurofisiología. Instituto de Biomedicina de Sevilla (IBiS), Hospital Universitario Virgen del Rocío/CSIC/Universidad de Sevilla, Seville, Spain; 20 Unidad de Trastornos del Movimiento, Servicio de Neurología y Neurofisiología. Instituto de Biomedicina de Sevilla (IBiS), Hospital Universitario Virgen del Rocío/CSIC/Universidad de Sevilla, Seville, Spain; 21 CIEN Foundation, Queen Sofia Foundation Alzheimer Center, Madrid, Spain; 22 Instituto de Salud Carlos III (ISCIII), Madrid, Spain; 23 BT-CIEN, Madrid, Spain; 24 Department of Molecular Neuropathology, Centro de Biología Molecular “Severo Ochoa” (CBMSO), Consejo Superior de Investigaciones Científicas (CSIC)/Universidad Autónoma de Madrid (UAM), Madrid, Spain; 25 Unidad de Demencias, Hospital Clínico Universitario Virgen de la Arrixaca, Madrid, Spain; 26 Fundación Pública Galega de Medicina Xenómica- CIBERER-IDIS, Santiago de Compostela, Spain; 27 Centro de Investigación Biomédica en Red de Diabetes y Enfermedades Metabólicas Asociadas, CIBERDEM, Madrid, Spain; 28 Hospital Clínico San Carlos, Madrid, Spain; 29 Department of Neurology, Hospital Universitario Son Espases, Palma, Spain; 30 Servei de Neurologia, Hospital Universitari i Politècnic La Fe, Valencia, Spain; 31 BIOMICs, País Vasco; Centro de Investigación Lascaray. Universidad del País Vasco UPV/EHU, Vitori-Gasteiz, Spain; 32 Neurology Service, Hospital Universitario La Paz (UAM), Madrid, Spain; 33 Hospital Donostia de San Sebastián, San Sebastián, Spain; 34 Fundación para la Formación e Investigación Sanitarias de la Región de Murcia, Murcia, Spain; 35 Servicio de Neurología-Hospital de Cabueñes-Gijón, Gijón, Spain; 36 Centro de Investigación y Terapias Avanzadas. Fundación CITA-alzheimer, San Sebastián, Spain; 37 Navarrabiomed, Pamplona, Spain; 38 Servicio de Neurología -Hospital Universitario Central de Asturias, Oviedo, Spain; 39 Barcelona βeta Brain Research Center – Fundació Pasqual Maragall, Barcelona, Spain; 40 Unitat de Genètica Molecular. Institut de Biomedicina de València-CSIC, Valencia, Spain; 41 Unidad Mixta de Neurologia Genètica. Instituto de Investigación Sanitaria La Fe, Valencia, Spain; 42 Hospital Universitario La Princesa, Madrid, Spain; 43 Hospital Clínic Barcelona, Barcelona, Spain

**Keywords:** Genomics, Psychiatric disorders

## Abstract

Long runs of homozygosity (ROH) are contiguous stretches of homozygous genotypes, which are a footprint of inbreeding and recessive inheritance. The presence of recessive loci is suggested for Alzheimer’s disease (AD); however, their search has been poorly assessed to date. To investigate homozygosity in AD, here we performed a fine-scale ROH analysis using 10 independent cohorts of European ancestry (11,919 AD cases and 9181 controls.) We detected an increase of homozygosity in AD cases compared to controls [*β*_AVROH_ (CI 95%) = 0.070 (0.037–0.104); *P* = 3.91 × 10^−5^; *β*_FROH_ (CI95%) = 0.043 (0.009–0.076); *P* = 0.013]. ROHs increasing the risk of AD (OR > 1) were significantly overrepresented compared to ROHs increasing protection (*p* < 2.20 × 10^−16^). A significant ROH association with AD risk was detected upstream the *HS3ST1* locus (chr4:11,189,482‒11,305,456), (β (CI 95%) = 1.09 (0.48 ‒ 1.48), *p* value = 9.03 × 10^−4^), previously related to AD. Next, to search for recessive candidate variants in ROHs, we constructed a homozygosity map of inbred AD cases extracted from an outbred population and explored ROH regions in whole-exome sequencing data (*N* = 1449). We detected a candidate marker, rs117458494, mapped in the *SPON1* locus, which has been previously associated with amyloid metabolism. Here, we provide a research framework to look for recessive variants in AD using outbred populations. Our results showed that AD cases have enriched homozygosity, suggesting that recessive effects may explain a proportion of AD heritability.

## Introduction

Alzheimer’s disease (AD) is a neurodegenerative disorder that is the leading cause of dementia worldwide^1^. AD presents a strong genetic component. Autosomal dominant mutations have been linked to familial early onset AD (EOAD) (<65 years): mutations in *presenilin 1* (*PSEN1*)^2^, *presenilin 2* (*PSEN2*)^3^, and *amyloid precursor protein* (*APP)*^4^. These findings lead to the role of amyloid metabolism as disease-causing mechanism^5^. Despite that, dominant causes account for a minority of both familial and sporadic EOAD cases, suggesting that autosomal recessive loci might cause most EOAD cases (∼90%)^6^. However, only two recessive mutations in the *APP* gene (A673V and E693Δ) have been described to date^7,8^, and this mode of inheritance for AD remains controversial.

The sporadic form of late-onset AD (LOAD) (>65 years) has a polygenic background. Heritability estimation for LOAD is, roughly, 70%^6^. Although, near to 40 loci has been associated with LOAD risk^9–12^, genetic variance captured by genome-wide strategies fall in a range of 7 –31%^9,13^, explaining a limited part of disease heritability. Current genetic findings were made using an additive mode of inheritance, which overlooks the relevance of non-additive genetic components, i.e., the recessive model. Despite the fact these components could explain a fraction of disease heritability.

It is well known that inbreeding increases the incidence of recessive diseases. The probability of detecting a recessive locus increases in offspring of consanguineous unions^14^, because the partners share alleles identical-by-descent. This recent parental relatedness points to genuine regions of autozygosity. Long runs of homozygosity (ROHs)—long stretches of consecutive homozygous genotypes (>1 Mb)—are a recognized signature of recessive inheritance and provide a measure of inbreeding in studied populations. Thus far, they have been used for homozygosity mapping^15^. Population history, e.g., historical bottlenecks or geographical isolation, also influences homozygosity levels in individual genomes^16,17^.

An excess of homozygosity has been associated with the risk of AD in individuals of Caribbean-Hispanic and African-American ancestries^18–20^, suggesting the presence of inbreeding and potentially autosomal recessive AD (arAD) cases nested in these populations. Conversely, this association was not replicated for individuals of European ancestry^21,22^. Several factors might explain these inconsistencies, among them it has been estimated that large sample sizes (12,000‒65,000) are required to detect an excess of homozygosity in outbred populations^23^. Thus, previous studies might be underpowered.

The limited number of deeply characterized consanguineous families, the difficulties in finding familial information for sporadic AD individuals (mainly due to the late onset of the disease) and the reduced size of intragenerational pedigrees in western countries make the search for recessive patterns of inheritance in AD complex. Furthermore, follow-up of candidate ROHs in sequencing data might be a necessary step in the definitive mapping of an arAD locus, but it has been poorly assessed to date.

Assessing the impact of homozygosity in the genetic architecture of AD, and subsequent follow-up of homozygous regions remains a challenge. To the best of our knowledge, this is the largest genomic data set exploring the influence of homozygosity in AD (*n* = 21,100). First, we investigated whether AD individuals from a European outbred population presented an excess of homozygosity relative to controls. Next, we measured the degree of inbreeding in AD cases. To prioritize regions with potential recessive loci, we constructed a homozygosity map of genomic regions overrepresented in detected inbred AD cases. Finally, we performed further exploration of several promising candidate ROHs using whole-exome sequencing (WES) data.

## Patients and methods

**Fig. 1:**
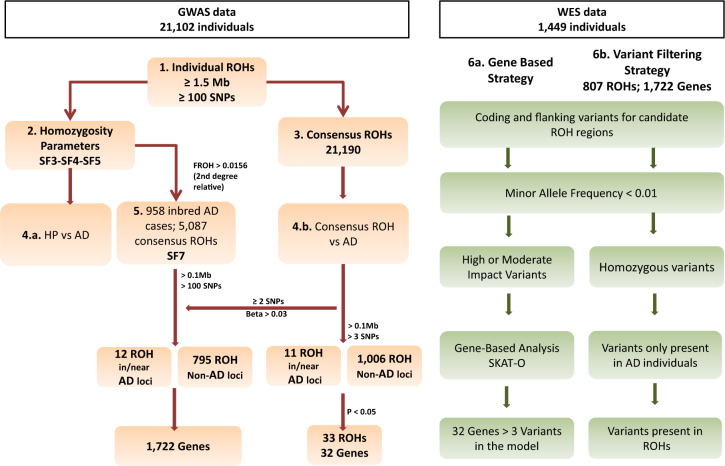
Schematic of the stepwise for ROH prioritization. 1. Identification of ROH segments per individual; 2. Estimation of: homozygosity parameters, and 3. Consensus ROHs; 4. Association analysis between: a) Homozygosity parameters and AD status, and b) Consensus ROH and AD status; 5. Identification of inbred AD cases and ROH prioritization; 6. Exploration of selected ROH segments in WES data applying: a) Gene-based strategy, and b) Variant filtering strategy.

The overview of the proposed strategy for ROH detection and subsequent prioritization is depicted in Fig. 1.

### Genotyping data

This study includes 10 independent genome-wide data sets comprising a total sample of 21,100 unrelated individuals (11,921 AD cases and 9181 individual controls) of European ancestry (Supplementary Table 1). The recruitment and phenotyping, has been described previously^12^.

Genotype-level data for each cohort was processed by applying identical quality control and imputation procedures, as previously reported^12^. Next, we generated a merged data set combining imputed genotypes (MAF > 0.05; imputation quality *R*^2^ > 0.90) from available data sets. We calculated identity-by-descendent (IBD) with PLINK 1.9 to generate a cohort of unrelated individuals of European ancestry (Supplementary Fig. 1). All possible pairs had Pi-hat < 0.1875, a Z0 ≥ 0.75 and a Z1 ≤ 0.25. Imputed markers with call rates >0.95 and MAF > 0.05 in the merged data set were selected for ROH calling (*N*_SNPs_ = 2,678,325).

### Runs of homozygosity (ROHs) exploration

#### 1-Identification of individual ROHs

Individual ROH calling was conducted using the observational genotype-counting approach implemented in PLINK (v1.09) (https://www.cog-genomics.org/plink/1.9/), as it outperforms additional methods in ROH detection and it is applicable to outbred populations^24^. ROH detection was performed for each individual study and for the merged data set using imputed genotypes. We used a sliding window of 50 SNPs of 5000 Kb in length to scan the genome. In order to manage genomic regions with a small number of genotyping errors and discrete missingness, one heterozygote and five missing calls per window were tolerated. These parameters were similar to those described previously^25^. The minimal number of SNPs in a ROH was set to 100 SNPs^26,27^. We empirically explored two minimal length cut-offs to consider a ROH, 1 Mb and 1.5 Mb. ROHs < 1.5 Mb might reflect LD patterns of ancient origin rather than the consanguineous cultural practices and genetic isolation captured with ROHs > 1.5 Mb^28^. SNPs were included in a ROH if >5% of the sliding window was homozygous. The maximum distance between two consecutive SNPs was set to 1000 Kb apart, and SNP density to at least 1 SNP in 50 Kb.

#### 2-Exploration of homozygosity parameters

To assess the data quality and genetic architecture of detected ROHs (>1 Mb and >1.5 Mb) in each individual study and in the whole dataset, we calculated: (a) the mean of the total length of ROH or sum of ROH (SROH); (b) the average ROH length (AVROH); (c) the number of ROHs (NROH); and (d) ROH-based estimates of the inbreeding coefficient, F, (FROH) per individual. AVROH is the SROH divided by NROH per subject. FROH represents the proportion of homozygous segments in the autosomal genome per individual (Eq. 1). For individuals, this would be the SROH detected divided by a factor of 3,020,190 Kb, the total autosomal genome length according to the GRCh37.p13 assembly. We further explored whether the effect of homozygosity parameters was similar when: (1) ROH length was set to 1 or 1.5 Mb; and (2) the analysis was performed per data set or in the final merged database. Results emerging from these exploratory analyses are shown in Supplementary Figs. 2–3, Supplementary Tables 2–3, and Supplementary Methods. According to them, we decided to conduct downstream analyses with ROH calling at 1.5 Mb in the merged data.

1 $${\it{F}}_{{\it{\mathrm{ROH}}}} = \frac{{\mathrm{SROH}\left( {\mathrm{Kb}} \right)}}{{\mathrm{Autosomal}}\,{\mathrm{genome}}\,\left( {\mathrm{Kb}} \right)}$$

Copy number variants (CNV), particularly hemizygous deletions, are known to cause spurious ROHs. However, prior studies have demonstrated that the impact of performing ROH calling with or without CNVs is only 0.3% of the total ROH length^28^. To assess the impact of CNVs deletions, we also conducted ROH calling after removing common CNV deletions extracted from the Database of Genomic Variants (DGV) (http://dgv.tcag.ca/)^29^. The same exercise was conducted after removing CNVs detected in GR@ACE dataset. Further description of CNV calling is provided in Supplementary material.

#### 3-Identification of consensus ROHs

Consensus ROHs were defined as overlapping segments between individual ROHs observed in different individuals, with DNA segment match of at least 95% for non-missing SNP markers. Consensus ROH calling was performed using PLINK 1.9. To prevent the detection of false-positive ROHs, we extracted those consensus ROHs with a length >100 Kb and >3 SNPs.

#### 4-Analyses

##### 4a-Association analysis between homozygosity parameters and AD risk

To assess the quality of the data in each individual study, we explored sample distribution for each of four homozygosity parameters: NROH, SROH, AVROH, and FROH. An exploratory analysis was depicted with violin plots, which combine a box plot with a kernel density plot, using the ggplot2 package from R (Supplementary Figs. 4 and 5). The inverse rank normal transformation was performed to generalize homozygosity parameters using “rankNorm” option in the RNOmni package in R. Transformed distributions are shown in Supplementary Fig. 6. To test the association of homozygosity parameters with AD status, we developed a generalized linear model for a binominal outcome, using R for individual-level data. We tested three models, adjusting per: (1) cohort and the first four principal components (PCs) resulting from ancestry analysis. See Eq. 2; (2) cohort, PCs and age; (3) cohort, PCs, age and gender. We also conducted a sensitivity analysis excluding control individuals <60 years old (See the “Results” section),

2 $$\begin{array}{l}Z = \beta _1{\mathrm{Homozygosity}}\,{\mathrm{Parameter}} + \beta _2{\mathrm{Cohort}}\\ + \beta _3{\mathrm{PC1}} + \beta _4{\mathrm{PC2}} + \beta _5{\mathrm{PC3}} + \beta _5{\mathrm{PC4}} + e\end{array}$$

##### 4b-Association analysis between consensus ROHs and AD

The association between the phenotype and consensus ROHs was explored using a logistic model, for ROHs in or near to previously identified AD loci extracted from de Rojas et al.^30^ and non-AD ROHs. The model was adjusted per cohort, and the first four PCs as covariates for downstream analysis. Covariate models adjusted for age and gender, in addition to cohort and PCs, were also calculated. Regression-based results were corrected for multiple testing using a Bonferroni correction.

Next, we sought to estimate whether there was an overrepresentation of risk (*β* > 0) or protective (*β* < 0) consensus ROHs in our association results at different levels of length and SNP number per consensus ROH. We applied a binominal test using R.

#### 5-The homozygosity map of inbred AD individuals

##### 5a-Identificationn of inbred individuals

We used F_ROH_ to detect the subset of inbred individuals within our dataset. F_ROH_ has been previously shown to better correlate with the unobserved pedigree inbreeding^23,31^. The cut-off between inbred and non-inbred individuals was set to *F*_ROH_ > 0.0156^32^, which corresponds to a second-degree relation. It was assumed that there are no different biological effects below 0.0156 than in the general population^33^. The efficient capture of inbred individuals is shown in Supplementary Fig. 7. Next, to explore whether the frequency of consanguinity was higher in AD cases than in controls, we calculated the odds ratio and chi square *p* values using the epitools package in R.

##### 5b-ROHs prioritization based on inbred AD cases

ROH detection was conducted in the subset of inbred AD cases, applying similar criteria to those previously described. Briefly, considering the long size of homozygous tracts for inbred individuals, there is a higher probability of finding a consensus ROH by chance within consanguineous AD cases than in the general population. Hence, we applied stringent criteria to define consensus ROHs. Consensus ROHs from inbred AD cases with ROH lengths >100 Kb and ROH > 100 SNPs were given priority for further analysis. Shared overlapping regions between inbred AD cases and the whole data set were also identified (See bash code in Supplementary Code Material) and selected based on their overrepresentation in AD cases relative to controls (*β* > 0.03). Prioritized regions were then explored in sequencing data. We also explored the overlapping of these regions with previously identified AD loci^30^.

### WES data

To meet the objective of exploring most promising ROH candidates in the sequencing data, we used the Knight-ADRC-NIA-LOAD (KANL) cohort^34^. We excluded autosomal dominant familial cases and sporadic AD cases harboring well-known disease-causing mutations, as they could explain disease status. Thus, this study comprised 986 AD cases and 463 control individuals of European ancestry (See Supplementary Table 1 and Supplementary Fig. 1). Of these, 488 subjects presented both GWAS and WES data available for this study. Detailed descriptions of cohort characteristics and quality control for WES data have been provided previously^34^.

#### 6-Candidate gene prioritization strategies using WES

##### 6a-Gene-based analysis

To prioritize genes in consensus ROH regions, we performed gene-based analysis (986 cases vs 463 controls) (Fig. 1). To generate variant sets, variants were filtered out according to minor allele frequency (MAF < 0.01) and functional impact. The allele frequency cut-off was established according to the Exome Aggregation Consortium (ExAC), non-Finnish European Exome Sequencing project (ESP), and 1000 G. Only those variants predicted to have a high or moderate effect according to SnpEff were included^35^. To compute *p*-values per gene set, SKAT-O model was applied using R. The models were adjusted to consider the impact of the first two PCs and sex. Genes were filtered out from results if the number of variants included in the model was ≤3.

##### 6b-Variant filtering strategy for inbred AD cases with WES and GWAS data available

ROH segments emerging from inbred AD cases are the most promising candidates to harbor autosomal recessive variants. Therefore, we deeply explored ROHs by applying a variant filtering strategy. We explored 488 AD cases with complementary GWAS and WES data. Because there is a low likelihood to identify any novel or causative mutation in available databases, variants with MAF > 0.01 were excluded. All heterozygous variants were removed. Finally, only the variants mapped in individual ROHs were selected.

To map genes within ROHs, we first extracted all the variants located in ROH regions. Next, we individually annotated each one.

## Results

### ROH parameters are associated with AD risk

**Fig. 2:**
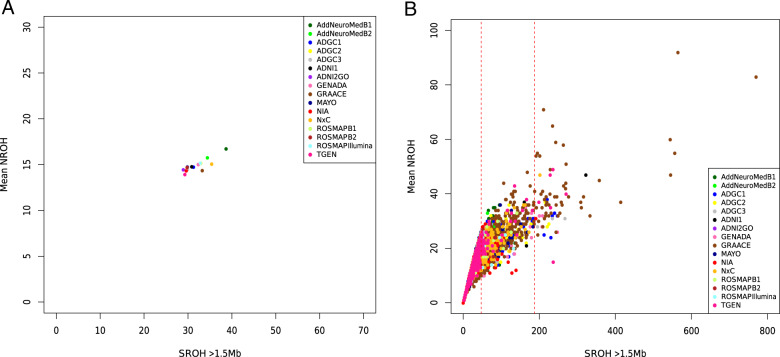
Runs of homozygosity per cohort and per individual. **A** Mean number of ROHs versus mean total sum of ROHs in Mb for the 10 cohorts explored. **B** Mean number of ROHs versus mean total sum of ROHs in Mb per individual explored. Red dashed lines represent the threshold for the inbreeding coefficient of 0.0156 (second cousins’ offspring) and 0.0625 (first cousins’ offspring).

We examined the typical characteristics of the four ROH parameters (SROH, NROH, AVROH, FROH) in 21,100 unrelated European individuals from 10 independent cohorts (Supplementary Tables 1–2 and Supplementary Fig. 4). Relationships between the mean NROH and SROH are shown in Fig. 2. The mean NROH was 14.6 ± 4.6, the AVROH was 2.11 ± 0.61 Mb, and the SROH was 31.9 ± 22.2 Mb. These estimations are in accordance with those observed in European individuals^32^, except for the NROH parameter, which was higher than in the previous studies^32^.

Next, we tested the association of the four homozygosity parameters with AD risk. We found that (i) higher inbreeding coefficient (*F*_ROH_) increased the risk of suffering AD [*β*_FROH_ (CI95%) = 0.043 (0.009–0.076); *p* value = 0.013] (Table 1); (ii) AD patients presented higher average lengths of ROHs compared to controls [*β*_AVROH_ (CI95%) = 0.07 (0.037–0.104); *p* value = 3.91 × 10^−5^]; (iii) ROH number was not associated with AD risk after adjusting for age [*β*_NROH_ (CI 95%) = 0.010 (−0.024–0.044); *p* value = 0.571] (Table 1). Results per cohort are shown in Supplementary Table 4. Notably, a sensitivity analysis conducted excluding: (1) known deletions, i.e., hemizygous segments^29^; and, (2) deletions identified in GR@ACE CNV study; provided comparable results (Supplementary Table 5). After excluding control individuals <60yo, a stable and significant effect remains for AVROH [*β*_AVROH_ (CI 95%) = 0.07 (0.031–0.103); *p* value = 3.51 × 10^−5^] (Supplementary Table 5).

**Table 1:** Effect of genome-wide homozygosity measures in Alzheimer’s disease for the joint analysis.

Dataset	Model 1		Model 2		Model 3	
	Beta (CI 95%)	*P* value	Beta (CI 95%)	*P* value	Beta (CI 95%)	*P* value
FROH	0.051 (0.023–0.078)	3.25 × 10^−4^	0.044 (0.010–0.077)	0.011	0.043 (0.009–0.076)	0.013
AVROH	0.027 (0.000–0.055)	0.051	0.074 (0.040–0.106)	2.16 × 10^−5^	0.070 (0.037–0.104)	3.91 × 10^−5^
NROH	0.043 (0.015–0.071)	2.48 × 10^−3^	0.010 (−0.024–0.044)	0.559	0.010 (−0.024–0.044)	0.571

Model 1: adjusted per Cohort and PCs; Model 2: Adjusted by cohort, PCs, and age; Model 3: Adjusted by cohort, PCs, age and gender.

Results for the association of excess of homozygosity (*F*_ROH_), average ROH lenght (AVROH), and number of ROH (NROH) with Alzheimer disease status.

OR, Odds ratio; with 95% confidence interval (CI 95%) and level of statistical significance (*P* value).

Association between homozygosity parameters and AD status, adjusted per Cohort, PCs, Age and Sex, was conducted in individuals with all available data; *N* = 19,253.

### ROH analysis of AD risk using the whole data set

We identified 21,190 consensus ROHs in the merged data set (*N* = 21,100). We observed a significant over-representation of ROHs increasing the risk of suffering AD (*p* value < 2.20 × 10^−16^) (Table 2). The same over-representation of risk associations was detected after filtering at several levels based on the length and number of SNPs per consensus ROH (Table 2). When the test was conducted with results adjusted for cohort, PCs, age, and gender, the over-representation of risk associations remained highly significant (*p* value < 2.20 × 10^−16^).

**Table 2:** Frequency of consensus ROHs with a potential risk or protective effect in Alzheimer’s disease.

	*N* ROH	Risk associations	Protective associations	P value	Probability of success
Whole dataset	21190	11974	9216	< 2.2 × 10^−16^	0.56
Category A	1017	593	424	< 2.2 × 10^−16^	0.58
Category B	926	537	389	1.30 × 10^−6^	0.57
Category C	858	499	359	1.98 × 10^−6^	0.58
Category D	42	33	9	2.7 × 10^−4^	0.79
Whole dataset/map of inbreed AD cases	6636	3969	2667	< 2.2 × 10^−16^	0.60

Strategy A, ROHs > 100 kb; >3 SNPs.

Strategy B, ROHs > 100 kb; >25 SNPs.

Strategy C, ROHs > 100 kb; >50 SNPs.

Strategy D, ROHs > 100 kb; >3 SNPs, *P* < 0.05.

We then tested the association of 11 consensus ROH (≥100 Kb and ≥3 SNPs) located in or near to previously identified AD loci (*N* = 38)^30^, with AD status (Supplementary Table 6). For these analyses, Bonferroni corrected significance threshold of *p* = 1.32 × 10^−3^ was pre-specified. We detected a strong association near to *HS3ST1* locus (consensus ROH length = 115.9 Kb; chr4:11,189,482‒11,305,456), (45 AD cases vs 12 controls, β (CI 95%) = 1.09 (0.48–1.48), *p* value = 9.03 × 10^−4^). This region survived age and gender adjustments (Supplementary Table 6), and was detected across 12 out of 16 datasets (Supplementary Table 7). The replication of this specific locus with AD, using ROH methodology, provides new insights of a potential recessive mechanism for this dementia locus. Among other ROH regions in or near to known AD loci (Supplementary Tables 6 and 10), we highlighted a 237 Kb ROH upstream the *APP* gene (chr21: 26,903,551–27,141,292), by its known role in AD^7,8,36^; detected in 38 AD cases vs 26 controls (26 vs 12 inbred individuals, respectively). For non-previously associated AD regions, none ROH (*N* = 1006) reached the significance threshold (Bonferroni correction of *p* = 4.97 × 10^−5^). Previous significant consensus ROH (chr8: 37835460–38143780) associated with AD in Europeans^21^ was not detected in this study, which is in line of results from Sims et al.^22^, failing replication.

We then explored the genes located in significant risk consensus ROHs (*p* value < 0.05) in gene-based analysis from WES data as well (Fig. 1). A total of 33 ROHs comprising 32 genes were analyzed (included > 3 SNPs in the model; Bonferroni correction *p* value = 0.0015). The *NECAB1* locus (chr8:91,803,921-91,971,630) presented the most significant signal (*p* = 0.01) (Supplementary Table 8), but none loci reached the multiple test correction threshold.

### Homozygosity mapping of AD using DNA segments identified in inbred cases

We detected 1621 individuals (958 Cases and 663 Controls) presenting a *F*_ROH_ ≥ 0.0156 among the total sample (*N* = 21,100) (Fig. 2) (Supplementary Table 9). Interestingly, inbreeding over the second degree of consanguinity was associated with a higher risk of suffering AD [OR (95%, CI) = 1.12 (1.01–1.25); *p* value = 0.027), which is in line with our previous results. This supports the idea that an excess of consanguineous individuals is present in the AD population. Accordingly, the search for recessive loci that play a role in AD can first be assessed in consanguineous cases.

**Fig. 3:**
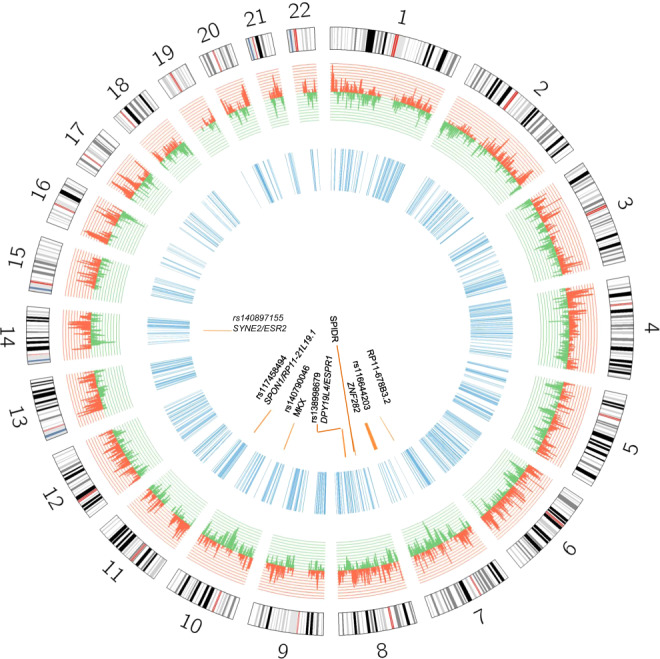
Circos plot for the prioritized regions. Histogram for the effect of the 21,190 consensus ROHs identified in the whole sample is shown. Risk ROH associations are shown in red; protective ROH associations are shown in green. Blue regions represent prioritized ROHs from consanguineous AD cases. Orange segments represent prioritized regions harboring potential recessive variants.

After ROH calling in inbred AD cases, we detected 5087 ROHs, and extracted those with ≥100 Kb and ≥100 SNPs. We then selected only over-represented regions in AD cases relative to controls in the general analysis (Fig. 1). We prioritized 807 consensus homozygous segments from inbred cases (8.6% of the total autosomal genome) (Fig. 3 and Supplementary Table 10). Among them, 12 ROHs were in or near to a previously identified AD loci (Supplementary Table 10).

After exploring genes in identified ROHs by gene-based analysis from WES data, none of them remained associated after multiple corrections (*N*_genes tested_ = 1136; *p* value = 3.47 × 10^−5^) (Supplementary Table 11). Our top signal was detected in the *FRY* locus (*p* value = 0.001) (Supplementary Table 11).

Considering that recessive variants are expected at very low frequencies, even gene-based analysis would be underpowered to detect significant associations. Therefore, we decided to further prioritize loci by searching homozygous mutations within selected consensus ROHs from inbred AD subjects (Fig. 1). We identified seven AD cases that had eight new (or extremely rare) homozygous variants within long ROH segments (Table 3). All ROH segments with homozygote variants were detected in more than 6 cohorts. Two of these individuals were consanguineous (*F*_ROH_ > 0.156). One had a missense variant (rs140790046, c.926A > G) that encodes p.Asn309Ser change within the *MKX* locus. Another carried a rare variant (rs116644203) in the *ZNF282* locus, which was in an extremely large region of homozygosity (14.9 Mb) (Table 3). Furthermore, three additional homozygous variants were detected: (i) a variant (rs117458494) in the *SPON1* locus, previously related with amyloid metabolism^37^, and (ii) two potential causative variants, carried only by this individual, within a previously identified AD region (*TP53INP/NDUFAF6)*^12^. One (rs73263258-*ESRP1*; in *TP53INP/NDUFAF6* region) is a missense variant (c.475G > A) that encodes p.Ala159Thr change (Table 3). Further notes and functional effect predictions for these variants are provided in Supplementary Table 12.

**Table 3:** Candidate recessive variants after ROH prioritization focused on inbred AD cases.

Individual	Froh _ROH>1500 kb_	SROH (Kb)	CHR	ROH start	ROH end	ROH lenght	ROH _SNPs_	Rs	Variant	Near locus	Ref Allele	Alt Allele	MAF	Impact	Variant Effect
1	0.0058	17421.6	7	53232690	55262627	2029.938	4213	NA	7:53930748	*RP11-678B3.2*	C	A	<0.0000	MODIFIER	non_coding_exon
2	0.0185	55861.4	7	135617715	150518393	14900.679	12426	rs116644203	7:148903805	*ZNF282*	C	T	<0.0000	LOW MODIFIER	synonymous sequence_feature non_coding_exon
3	0.0099	29868.8	8	47018484	49544784	2526.301	1265	NA	8:48352954	*SPIDR*	T	A	0.004	MODERATE LOW MODIFIER	missense sequence_feature 3_prime_UTR upstream_gene non_coding_exon
4	0.0153	46194.5	8	91282507	96735572	5453.066	4734	rs73263258	8:95658495	ESPR1*	G	A	0.002	MODERATE MODIFIER	missense 5_prime_UTR non_coding_exon
								rs138998679	8:95780656	DPY19L4*	A	G	0.003	LOW	synonymous
5	0.0179	54137.7	10	24516308	28414933	3898.626	6248	rs140790046	10:27964291	*MKX*	T	C	0.0005	MODERATE	missense
6	0.0137	41392.6	11	12032272	16358722	4326.451	4473	rs117458494	11:14282085	*SPON1* *RP11-21L19.1*	G	A	<0.0000	MODIFIER	upstream_gene downstream_gene intronic
7	0.0115	34603.4	14	63906601	66333670	2427.07	2633	rs140897155	14:64688393	*SYNE2* *ESR2*	G	A	0.001	MODERATE LOW MODIFIER	missense sequence_feature 3_prime_UTR downstream_gene intronic non_coding_exon_

MAF < 0.0000; Variants with non reported allele frequency in ExAC, ESP, 1000G reference database.

## Discussion

This study represents the largest analysis of homozygosity conducted for AD. Our estimates of homozygosity provide a robust evidence supporting that recessive allelic architecture might define a portion of AD heritability.

Previous AD ROH studies in European populations have shown negative results for the association of ROH parameters with AD^21,22^. First studies had very modest sample sizes (*N* < 3000, vs *N*_present study_ = 21,100)^21,22^, and likely were underpowered. Then, these studies used a ROH calling lengths set to 1 Mb^21,22^. This generates substantial inflation in the inbreeding coefficient (*F*_ROH_) and makes undetectable the enrichment in consanguinity due to unspecific noise (Supplementary material). These reasons might explain initial failures. We encourage other groups to conduct ROH analysis in new unrelated populations, but with large enough sample sizes and redefining the ROH lengths at least to 1.5 Mb, to better capture the recessive component of AD.

At the present study we identified a study-wise significant ROH association close to the *HS3ST1* gene (~200 Kb). Genetic markers near to this ROH (~300 kb) have been previously associated with AD using additive models^38,39^, and *HS3ST1* locus was differentially expressed in the brain of AD cases versus controls^38^. Our finding reinforces the association of this region with AD, and further suggests the role of recessiveness in explaining underlying associations. High-resolution mapping across this ROH could help to identify the causative mutation.

This study failed replication of previously detected ROH at chr8:37835460–38143780^21^. Although, both studies include TGEN cohort, overlapping to some extent, the default technical parameters for ROH definition were completely different (ROH calling: 1 Mb vs 1.5 Mb). We assume that technical differences of the present study respect to prior ones, might be critical points impacting replication of ROH findings, in addition to other causes, e.g. population-specific genetic patterns, or, even, random chance.

A strength of the present study comes from our effort to prioritize consensus ROHs according to the homozygosity map of inbred AD individuals, performed by the first time in AD, and our capacity to explore them in sequencing data. This strategy lets us to find interesting candidate recessive variants in: *MKX* and *ZNF282* genes, identified in two independent inbred AD cases; *TP53INP1/NDUFAF6* genomic region, previously associated with AD^12,40^; and *SPON1* locus. The *SPON1* locus deserves a further explanation as it is directly related with APP metabolism, a key player in AD physiopathology. APP cleavage through β-secretases produces amyloid-beta (Aβ), which later accumulates in AD brains^5^. SPON1 has been found to bind to APP, inhibiting its α/β cleavage^37^, and to APOE family of receptors^41^. Markers in this gene have been related to dementia severity^42^ and with the rate of cognitive decline^43^. Considering prior findings and the present result, it would be biologically plausible that the presence of recessive variants in *APP*^7,8^, or its biological partners directly influences the amyloid cascade. Thus, we believe that *SPON1* could be considered an interesting candidate, which deserves future resequencing efforts.

Our observations are subject to limitations that need to be considered. Data sets used in this study were genotyped using different platforms and shared a small proportion of directly genotyped markers. Given that lower SNP density could impact the accuracy of the study^32^, high quality imputed markers were used (*r*^2^ > 0.90, MAF > 0.05). Second, to reduce dataset heterogeneity we use a set of European individuals; applied the same GWAS quality control per study; generated a merged dataset including common variants across datasets; and controlled all our analyses by cohort, to account with potential confounding.

We assumed that differences in the ROH parameters between the cases and controls are modest. In that sense, we are not expecting a very large percentage of recessive AD cases, but we expect a fraction, in the same way, that it occurs for autosomal dominant forms (<1%). Considering that, the reported findings are supporting the hypothesis of this work, a group of recessive mutation may explain a portion of AD cases. However, we suspect that the existence of a large non-allelic heterogeneity is preventing its identification.

Our gene-based analysis strategy did not show significant associations. With a decreasing allele frequency and high locus heterogeneity, the power to detect genes of interest also decreases. Despite our effort to include WES data in the present study, the available sample size could be underpowered.

The potential impact of CNV deletions on ROH analysis must be taken into consideration. Thus, we assessed its effect on our analyses, but no differences were found before and after CNV exclusion (Supplementary Table 5), which is in agreement with the previous studies^25^. Clonal mosaicism, due to aging^44^, could also generate spurious ROHs. At the present study an age-dependent increase in the NROH was detected in the control group (Supplementary Table 13), which partially disappeared after excluding consensus ROHs associated with age (*p* < 0.05) (See Supplementary Material, and Supplementary Table 13 and 14). We assumed that these DNA segments might contain somatic alterations, confounding ROH associations. Among age-related ROH regions, we identified some loci previously associated with AD, e.g., *RORA*, *CD2AP*, *HS3ST1*, and amyloid-beta burden, e.g., *GLIS3*^45^; suggesting that some known AD regions could be affected by this phenomenon. These findings deserve future investigations. Despite the existence of ROH segments associated to age and somatic mosaicism phenomena, our most significant findings largely supported adjustments by age. Therefore, we feel that the major observations of this study are not affected by age-related instability of the human genome.

In summary, we demonstrated the existence of an inbreeding effect in AD and efficiently captured a fraction of inbred individuals from outbred populations, providing an improved strategy to look for recessive alleles, and to conduct future large-scale homozygosity mapping studies in AD. Furthermore, the exploration of complementary sequencing data gave an added value to this research, providing a subset of potential candidates harboring recessive variants. In any case, the proposed candidates would need confirmation in larger series. Greater efforts and larger collections of individuals with GWAS and sequencing data are needed to confirm the present findings.

Our understanding of the dynamics of population genomics in AD is far from complete, but ROH analyses provide us with a means to go further and might be an alternative strategy to uncover the genetic loci underlying AD.

## Supplementary information

Supplementary Material

Supplementary Figure 1

Supplementary Figure 2

Supplementary Figure 3

Supplementary Figure 4

Supplementary Figure 5

Supplementary Figure 6

Supplementary Figure 7

Supplementary Tables

Supplementary Code

## Data Availability

Script used to identify shared overlapping regions between inbred AD cases and the whole data set is available at Supplementary Code Material. Additional scripts used to conduct ROH calling are available from the authors upon request.

## Data availability

GR@ACE datased analysed during the current study is available into the European Genome-phenome Archive (EGA) (https://ega-archive.org), which is hosted by the EBI and the CRG, under accession number EGAS00001003424 (https://ega-archive.org/studies/EGAS00001003424). Additional GWAS datasets used in these study are available upon request in dbGaP. WES dataset used in the study can be downloaded from https://dss.niagads.org/studies/sa000004/. Data used in preparing this article were obtained from the Alzheimer’s Disease Neuroimaging Initiative (ADNI) database (adni.loni.usc.edu). As such, the investigators within the ADNI contributed to the design and implementation of ADNI and/or provided data but did not participate in the analysis or writing of this report. A complete listing of ADNI investigators can be found at http://adni.loni.usc.edu/wp-content/uploads/how_to_apply/ADNI_Acknowledgement_List.pdf.
