# Supplementary material for: Long runs of homozygosity are associated with Alzheimer’s disease
Source: Transl Psychiatry. 2021 Feb 24;11:142. doi: 10.1038/s41398-020-01145-1 (PMC7904832; doi:10.1038/s41398-020-01145-1)
Supplement: Supplementary file 1 — Supplementary Material [file 41398_2020_1145_MOESM1_ESM.docx]

**Supplementary Material**

# Tittle

**Long Runs of Homozygosity are associated with Alzheimer’s disease**

# **Authors**

Sonia Moreno-Grau PhD^1,2^, Maria Victoria Fernández PhD^3,4^, Itziar de Rojas M.Sc. ^1,2^ Pablo Garcia-González^1^, Isabel Hernández MD PhD^1^, Fabiana Farias PhD^3,4^, John P Budde^3,4^, Inés Quintela PhD^5^, Laura Madrid^6^, Antonio González-Pérez PhD^6^, Laura Montrreal^1^, , Emilio Alarcón-Martín ^1^, Montserrat Alegret PhD^1^, Olalla Maroñas PhD^5^, Juan Antonio Pineda MD^7^, Juan Macías MD^7^, GR@ACE & DEGESCO consortia, Alzheimer’s Disease Neuroimaging Initiative*, Marta Marquié MD PhD^1,2^, Sergi Valero PhD^1,2^, Alba Benaque^1^, Jordi Clarimón PhD ^2,8^, Maria Jesus Bullido PhD^2,9,10^, Guillermo García-Ribas MD^11^, Pau Pástor MD PhD^12^, Pascual Sánchez-Juan MD PhD^2,13^, Victoria Álvarez PhD^14,15^, Gerard Piñol-Ripoll MD PhD^2,16^, Jose María García-Alberca MD^17^, José Luis Royo PhD^18^, Emilio Franco-Macías MD^19^, Pablo Mir MD^2,20^, Miguel Calero PhD^2,21,22^, Miguel Medina PhD^2,21^, Alberto Rábano PhD^2,21,23^, Jesús Ávila PhD^2,24^, Carmen Antúnez MD ^25^, Luis Miguel Real PhD^7, 18^, Adelina Orellana PhD^1^, Ángel Carracedo PhD^5,26^, María Eugenia Sáez PhD^6^, Lluis Tárraga M.Sc^1,2^, Mercè Boada MD PhD^1,2^, Carlos Cruchaga PhD ^3,4^, Agustín Ruiz MD PhD^1,2**^

# Affiliation

1. *Research Center and Memory clinic Fundació ACE. Institut Català de Neurociències Aplicades. Universitat Internacional de Catalunya, Barcelona, Spain*
2. *CIBERNED, Center for Networked Biomedical Research on Neurodegenerative Diseases, Carlos III Institute of Health, Spain*
3. *Department of Psychiatry, Washington University School of Medicine, St. Louis, MO, United States of America*
4. *Hope Center for Neurological Disorders, Washington University School of Medicine, St. Louis, MO, United States of America*
5. *Grupo de Medicina Xenómica, Centro Nacional de Genotipado (CEGEN-PRB3-ISCIII). Universidade de Santiago de Compostela, Santiago de Compostela, Spain*
6. *CAEBI. Centro Andaluz de Estudios Bioinformáticos, Sevilla, Spain*
7. *Unidad Clínica de Enfermedades Infecciosas y Microbiología. Hospital Universitario de Valme, Sevilla, Spain*
8. *Memory Unit, Neurology Department and Sant Pau Biomedical Research Institute, Hospital de la Santa Creu i Sant Pau, Universitat Autònoma de Barcelona, Barcelona, Spain*
9. *Centro de Biologia Molecular Severo Ochoa (C.S.I.C.-U.A.M.), Universidad Autonoma de Madrid, Madrid, Spain*
10. *Instituto de Investigacion Sanitaria "Hospital la Paz" (IdIPaz), Madrid, Spain*
11. *Hospital Universitario Ramón y Cajal, Madrid, Spain*
12. *Fundació per la Recerca Biomèdica i Social Mútua Terrassa, and Memory Disorders Unit, Department of Neurology, Hospital Universitari Mutua de Terrassa, University of Barcelona School of Medicine, Terrassa, Barcelona, Spain*
13. *Neurology Service “Marqués de Valdecilla” University Hospital (University of Cantabria and IDIVAL), Santander, Spain*
14. *Laboratorio de Genética Hospital Universitario Central de Asturias, Oviedo*
15. *Instituto de Investigación Biosanitaria del Principado de Asturias (ISPA)*
16. *Unitat Trastorns Cognitius, Hospital Universitari Santa Maria de Lleida, Institut de Recerca Biomédica de Lleida (IRBLLeida), Lleida, España*
17. *Alzheimer Research Center & Memory Clinic, Andalusian Institute for Neuroscience, Málaga, Spain*
18. *Dep. of Surgery, Biochemistry and Molecular Biology, School of Medicine. University of Málaga, Málaga, Spain*
19. *Unidad de Demencias, Servicio de Neurología y Neurofisiología. Instituto de Biomedicina de Sevilla (IBiS), Hospital Universitario Virgen del Rocío/CSIC/Universidad de Sevilla, Seville, Spain*
20. *Unidad de Trastornos del Movimiento, Servicio de Neurología y Neurofisiología. Instituto de Biomedicina de Sevilla (IBiS), Hospital Universitario Virgen del Rocío/CSIC/Universidad de Sevilla, Seville, Spain*
21. *CIEN Foundation, Queen Sofia Foundation Alzheimer Center, Madrid, Spain*
22. *Instituto de Salud Carlos III (ISCIII), Madrid, Spain*
23. *BT-CIEN*
24. *Department of Molecular Neuropathology, Centro de Biología Molecular "Severo Ochoa" (CBMSO), Consejo Superior de Investigaciones Científicas (CSIC)/Universidad Autónoma de Madrid (UAM)*
25. *Unidad de Demencias, Hospital Clínico Universitario Virgen de la Arrixaca*
26. *Fundación Pública Galega de Medicina Xenómica- CIBERER-IDIS, Santiago de Compostela, Spain*

****Corresponding author**: Agustín Ruiz M.D. Ph.D.

**Address:** Research Center. Fundació ACE. Institut Català de Neurociències Aplicades. C/ Marquès de Sentmenat, 57, 08029 Barcelona, Spain

**Tel:** +3493.444.73.18

**Fax:** +3493.410.17.01

**Email id**: aruiz@fundacioace.org

**Conflict of Interest:** None.

**Alzheimer’s Disease Neuroimaging Initiative**: Data used in preparing this article were obtained from the Alzheimer’s Disease Neuroimaging Initiative (ADNI) database (adni.loni.usc.edu). As such, the investigators within the ADNI contributed to the design and implementation of ADNI and/or provided data but did not participate in the analysis or writing of this report. A complete listing of ADNI investigators can be found at http://adni.loni.usc.edu/wp-content/uploads/how_to_apply/ADNI_Acknowledgement_List.pdf

# Material and Methods

## Exploration of homozygosity parameters

We further explored whether the effect of homozygosity parameters was similar when: 1) ROH length was set to 1 Mb or 1.5 Mb; and 2) the analysis was performed per data set or in the final merged database (Supplementary Figure 2) Supplementary Table 2 and Supplementary Figure 3 demonstrate that FROH estimates derived from ROH calling at 1Mb exhibited a large degree of inflation, not allowing an accurate detection of inbreeding (Mean FROH 1Mb = 0.028; Mean FROH 1.5Mb = 0.011), in accordance with prior studies (34). After conducting an analysis of the 2,678,325 SNPs shared between available data sets, we found that the parameters of the individual data sets and the merged data set analyses were similar (Supplementary Figure 2 and Supplementary Table 3). After these exploratory analyses, we decided to conduct downstream analyses with ROH calling at 1.5 Mb in the merged data.

**CNV calling and QC in Gr@ACE cohort**

We used the PennCNV-Affy program and followed the analysis described by PennCNV website to complete CNV calling (<http://penncnv.openbioinformatics.org/en/latest/user-guide/affy/>) (1). First, we generated canonical genotype clustering files from the above files, e.g, generate_affy_geno_cluster.pl --nopower2 -locfile Axiom_SpainBA.na35.annot.db-probemappings.txt -sexfile GRACE1.5.sex.v1 -out GRACE1.5.genocluster AxiomGT1.calls.txt AxiomGT1.confidences.txt AxiomGT1.summary.txt. As the signal intensity values had not been log2 normalized, the –nopower2 argument was used, as previously described (2). Next, we calculated Log R Ratio (LRR) and B-Allele Frequency (BAF) values using the *normalize_affy_geno_cluster.pl* command, e.g.: normalize_affy_geno_cluster.pl batch1.genocluster AxiomGT1.summary.txt -nopower2 -locfile mapfileAX.dat -out dataset_lrr_baf.txt Finally, for CNV calling by PennCNV, we first splited the signal files into individual files and then detected the raw CNV calls. We created pfb and gcmodel files from existing data and “trained” a .hmm file on 100 Axiom samples, using the available Affy 6.0 hmm file as a template.

To assess the quality controls of generated CNVs, we explored distribution of QC parameter and removed individuals from the study if: they had >30 CNVs, had a genotype call rate <97%, had a waviness factor (WF) >0.0065 or <-0.0065, and LRR SD > 0.46.

**Age related ROHs regions**

To investigate whether potential age associated regions, which could be introduced by clonal mosaicism events, are disturbing ROH results, we first identify consensus ROHs associated with age using a logistic model in R. We explored three scenarios: 1) Model A includes cases and controls, and was adjusted by PCs, cohort and status; 2) Model B only includes case, and was adjusted by PCs and cohort; and 3) Model C only includes controls, and was adjusted by PCs and cohort. Then, we exclude those regions (±250 Kb) showing, at least, a nominal significance (p value < 0.05) in any of the three scenarios, re-calculate ROH parameters, and their correlation with age.

To discard that results emerging from this analysis were caused by a reduction in the number of SNPs, instead of by the exclusion of age related regions, we repeated x100 the same analysis described above but excluding 3,339 random non-age related ROHs. We detected that the chance to obtain a non-significant correlation with age for SROH, AVROH and NROH was 0% in all cases.

# References

1. Wang, K. et al. PennCNV: an integrated hidden Markov model designed for high-resolution copy number variation detection in whole-genome SNP genotyping data. *Genome Res* **17**, 1665–74. (2007)

2. Kendall, KM. et al. Cognitive Performance Among Carriers of Pathogenic Copy Number Variants: Analysis of 152,000 UK Biobank Subjects. *Biol Psychiatry*. **82,** 103–110, (2017)

# Supplementary Tables Legend

**Supplementary Table 1.** Characteristics of the cohorts used in the analysis.

**Supplementary Table 2.** Summary of homozygosity measures for each individual study and the merged data set, considering two minimal ROH length cut-offs, 1 Mb and 1.5 Mb.

**Supplementary Table 3.** Summary statistics for the difference in homozygosity measures calculated using two different methods

**Supplementary Table 4**. Effect of genome-wide homozygosity measures in Alzheimer’s disease for each individual data set

**Supplementary Table 5.** Effect of genome-wide homozygosity measures in Alzheimer’s disease for the joint analysis, excluding deletions.

**Supplementary Table 6**. Consensus ROHs associated with Alzheimer’s disease in the whole dataset.

**Supplementary Table 7.** Number of carriers of the study-wide significant ROH at chr4: 11,189,482‒11,305,456 per dataset.

**Supplementary Table 8.** Gene-based results for genes located in consensus ROHs associated with Alzheimer’s disease in the whole dataset.

**Supplementary Table 9.** Demographics for the pool of inbred individuals.

**Supplementary Table 10.** Consensus prioritized ROH based on the map of inbred Alzheimer's disease patients.

**Supplementary Table 11**. Gene-based results for genes located in consensus prioritized ROH based on the map of inbred Alzheimer's disease patients.

**Supplementary Table 12.** Variant annotation and functional effect prediction.

**Supplementary Table 13.** Effect of genome-wide homozygosity parameters in Alzheimer Disease for the joint analysis considering the effect of clonal mosaicism in aged populations.

**Supplementary Table 14.** Age associated consensus ROHs detected in Case/Control, Case only and control only studies.

# Supplementary Figure Legend

**Supplementary Figure 1.** Quality control for A) ancestry analysis in GWAS data, relatedness ancestry analysis in WES data. All possible pairs had Pi-hat < 0.1875, Z0 ≥ 0.75 and Z1 ≤ 0.25.

**Supplementary Figure 2.** Boxplot for FROH per individual at ROH calling with 1Mb and 1.5Mb. Red line represents FROH = 0.0156 (mean inbreeding coefficient for kinship of second cousin marriage).

**Supplementary Figure 3.** Mean number of ROHs versus mean total sum of ROHs in Mb for the 10 cohorts explored, according to different ROH calling parameters. A) ROH length set to 1 Mb. ROH calling conducted with different number of markers per data set; B) ROH length set to 1.5 Mb. ROH calling conducted with different number of markers per dataset; C) ROH length set to 1 Mb. ROH calling conducted with the fraction of markers shared between data sets (2.6M); D) ROH length set to 1.5 Mb. ROH calling conducted with the fraction of markers shared between data sets (2.6M).

**Supplementary Figure 4.** Violin plots showing the distribution of ROH > 1.5 Mb within each data set and in the merged data for the homozygosity parameters (NROH, SROH, AVROH, FROH).

**Supplementary Figure 5.** Violin plots showing the distribution of ROH > 1.5 Mb within each data set and in the merged data for the homozygosity parameters (NROH, SROH, AVROH, FROH), split by case control status.

**Supplementary Figure 6.** Transformed distribution for the homozygosity measures. Transformation was performed using an inverse rank normal transformation with the “rankNorm” option in the RNOmni package in R.

**Supplementary Figure 7**. Distribution for average length of individual ROH segments for: non-inbred individuals (FROH < 0.0156), second-degree relatives (FROH > 0.0156) and first-degree relatives (FROH > 0.0625).
