# Supplementary figures and images for: Long runs of homozygosity are associated with Alzheimer’s disease
Source: Transl Psychiatry. 2021 Feb 24;11:142. doi: 10.1038/s41398-020-01145-1 (PMC7904832; doi:10.1038/s41398-020-01145-1)

1A.

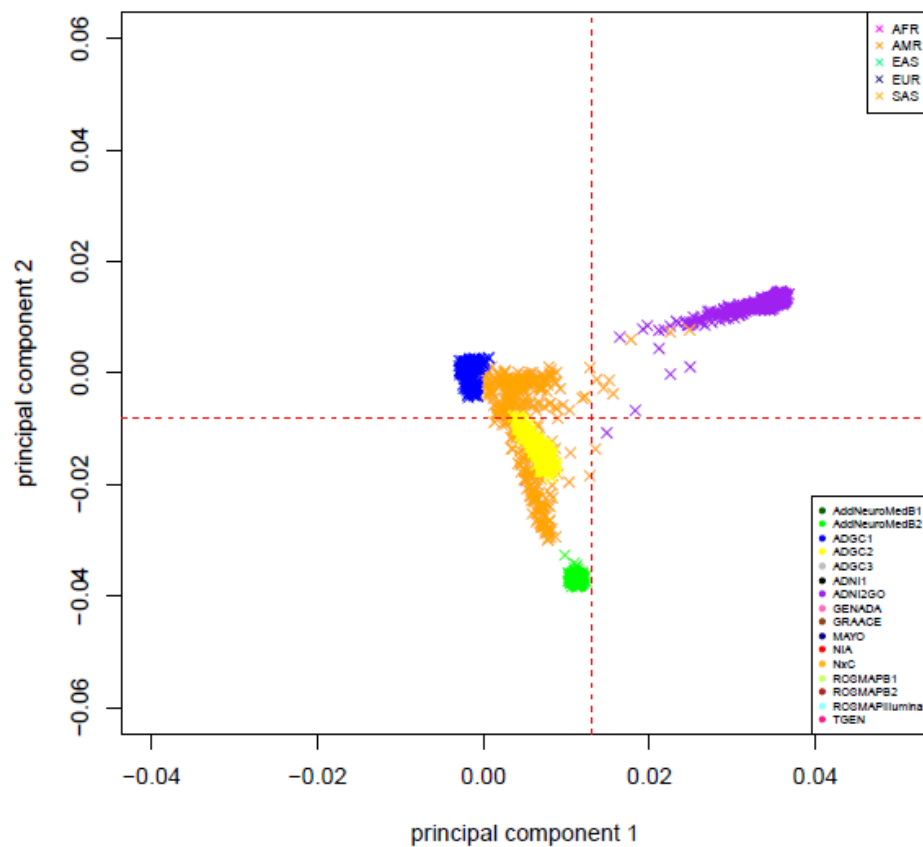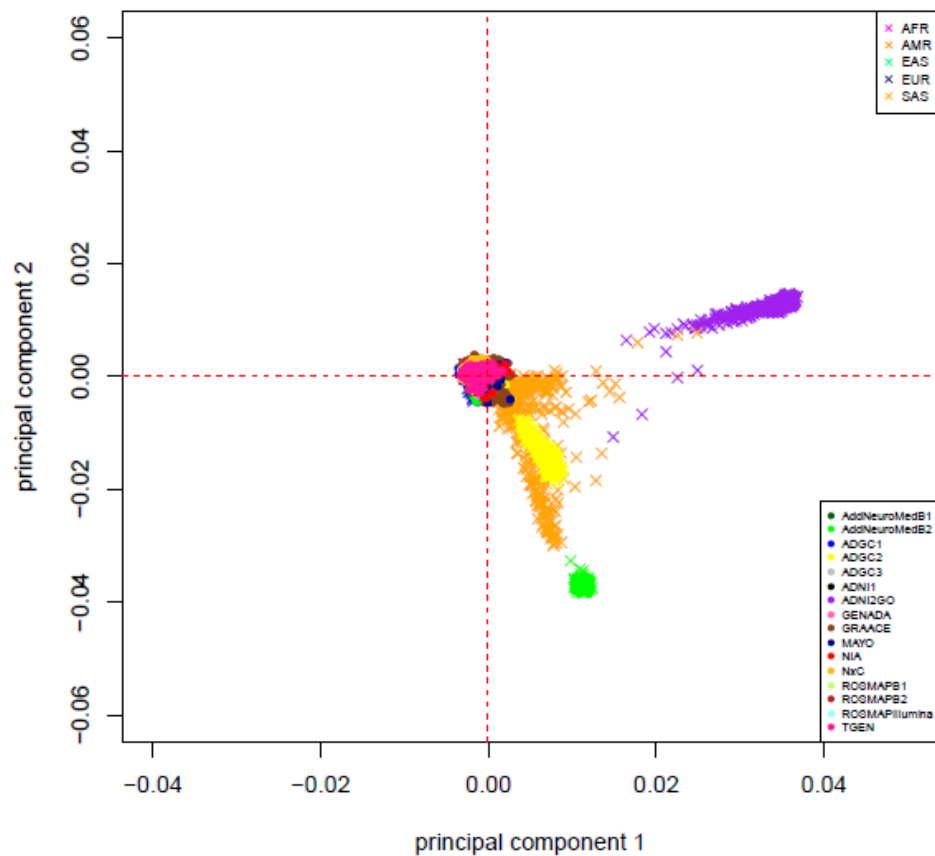

1B.

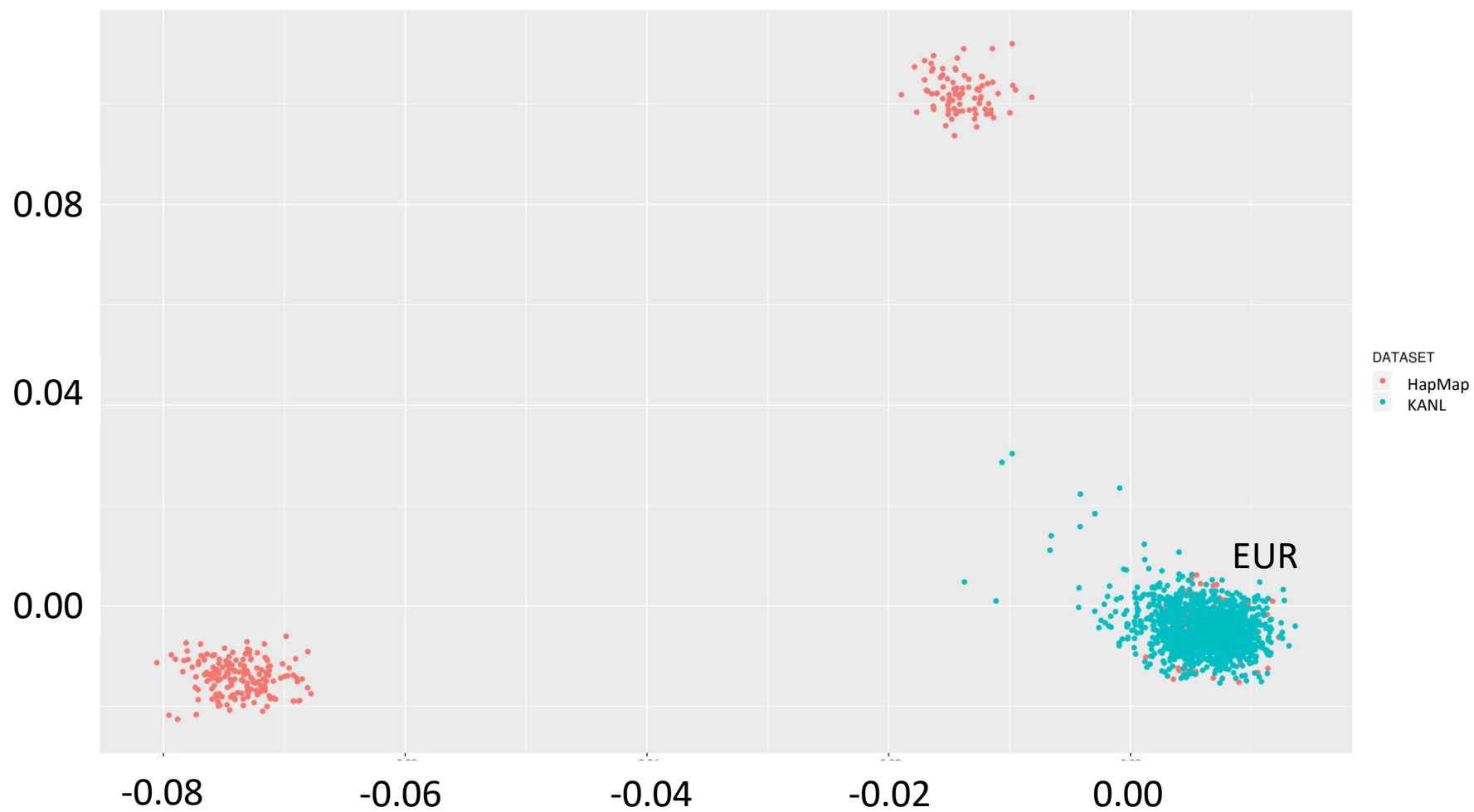

1C.

Z1

1.00

0.75

0.50

0.25

0.00

0

0.25

0.50

0.75

1.00

Z0

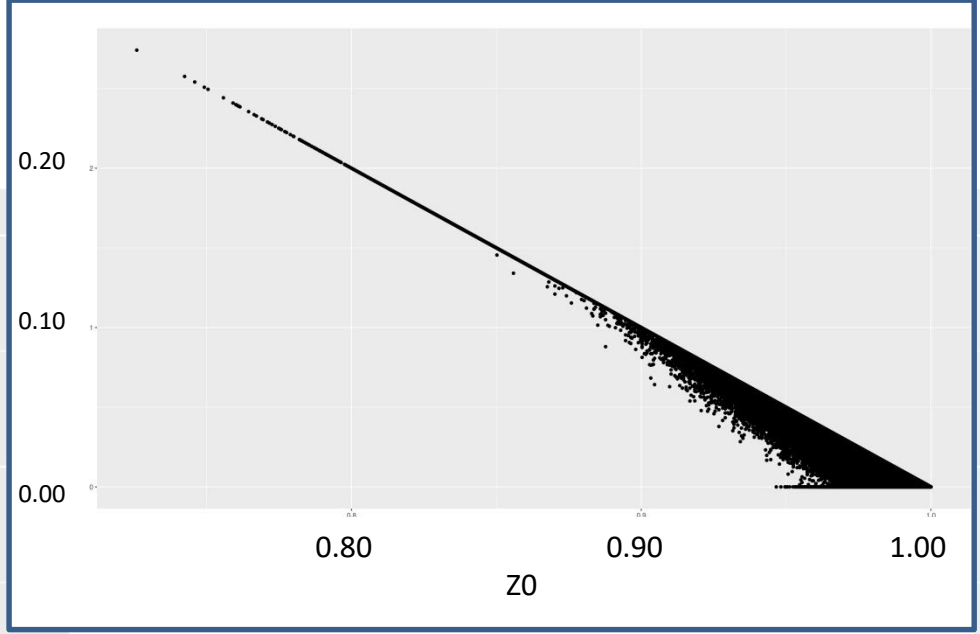

Supplement: Supplementary file 2 — Supplementary Figure 1 [file 41398_2020_1145_MOESM2_ESM.pdf]

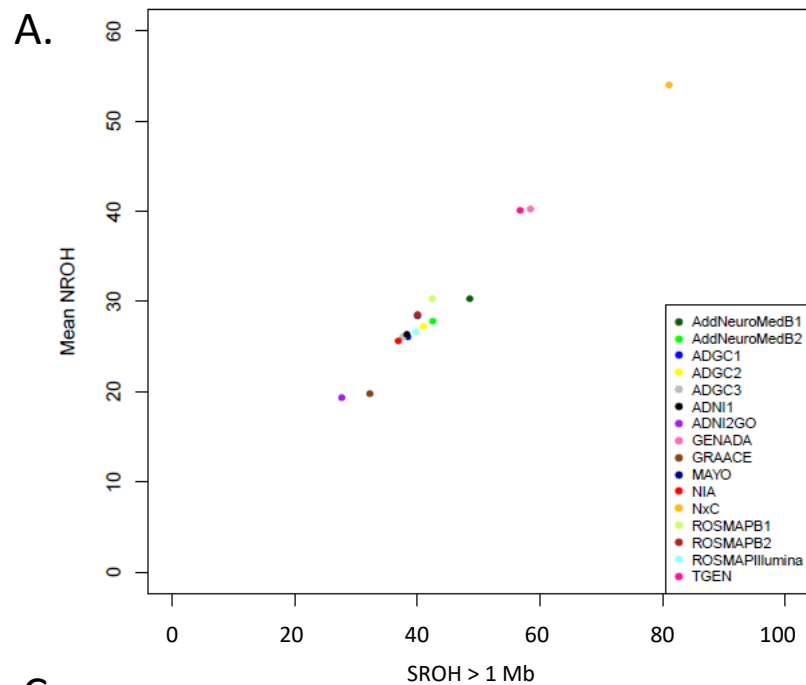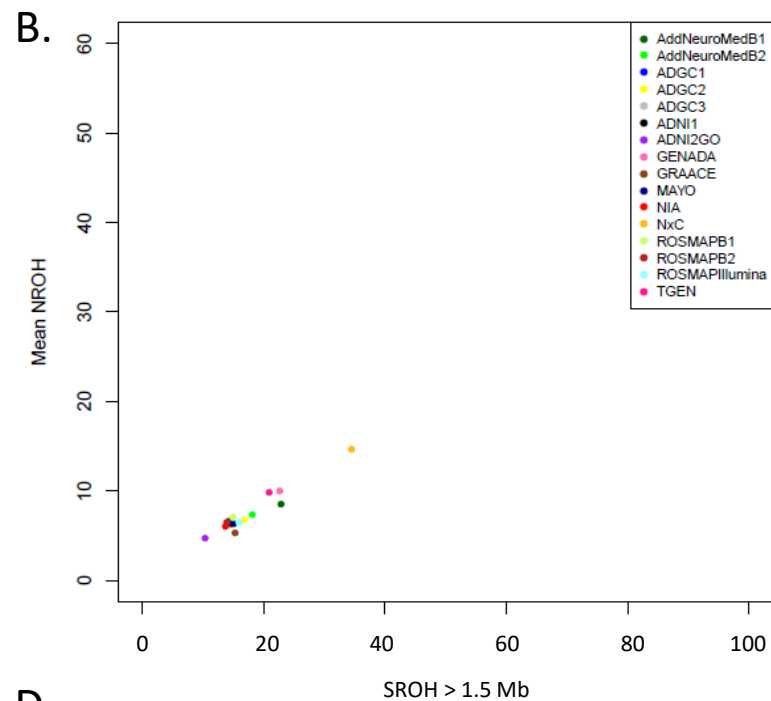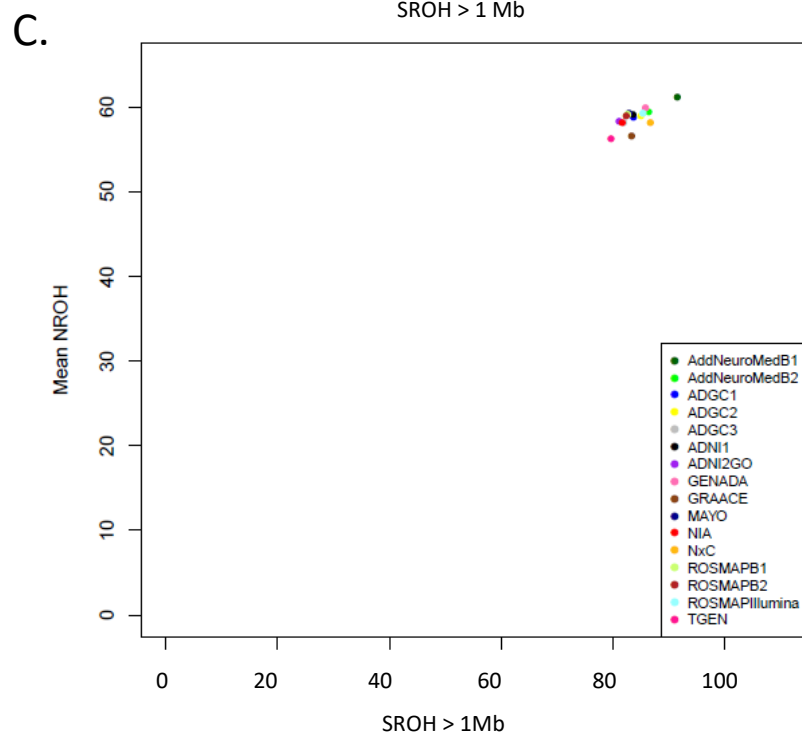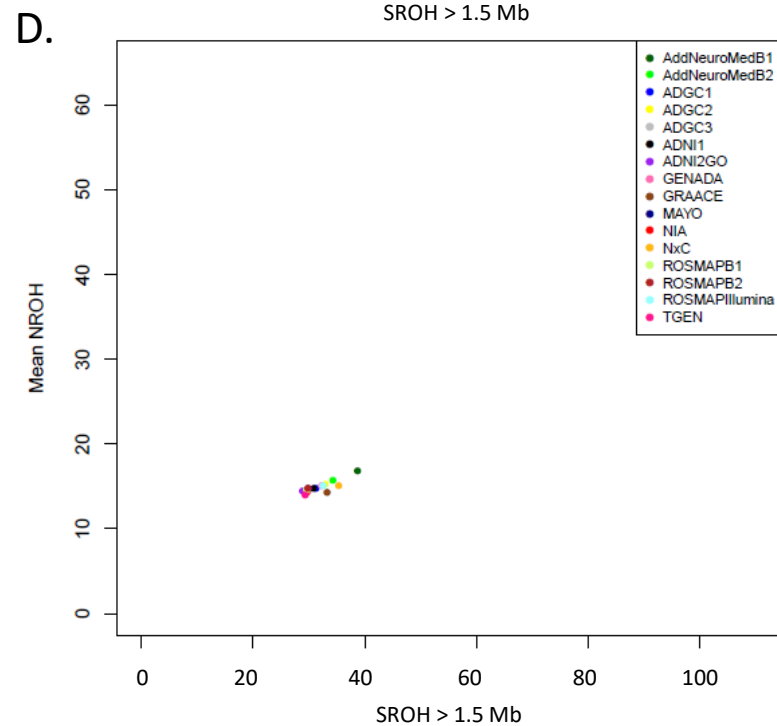

Supplement: Supplementary file 3 — Supplementary Figure 2 [file 41398_2020_1145_MOESM3_ESM.pdf]

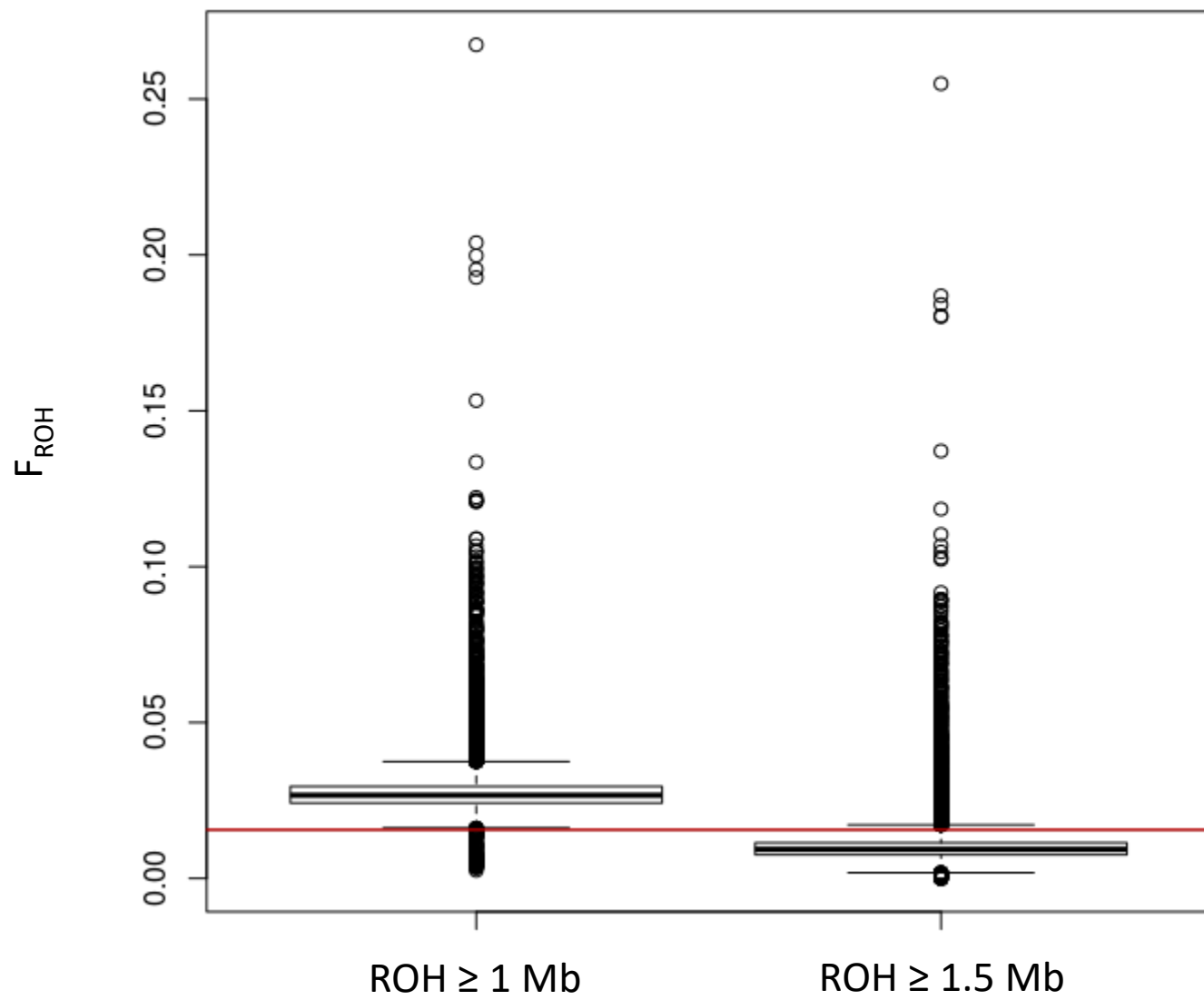

Supplement: Supplementary file 4 — Supplementary Figure 3 [file 41398_2020_1145_MOESM4_ESM.pdf]

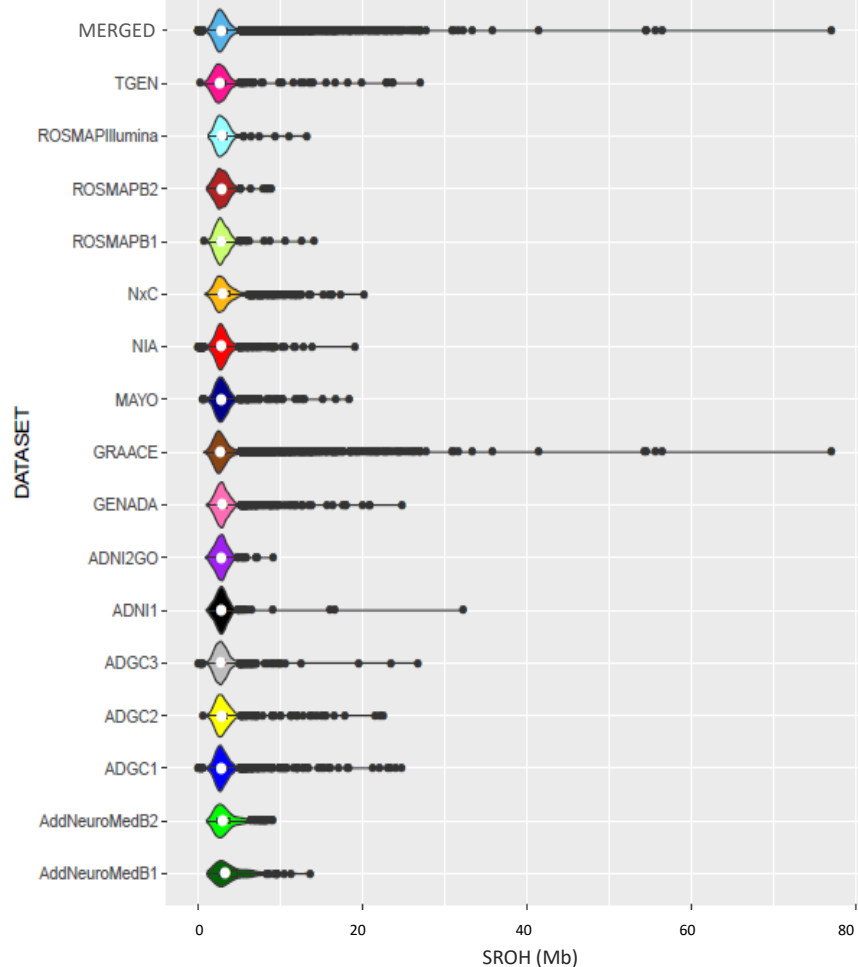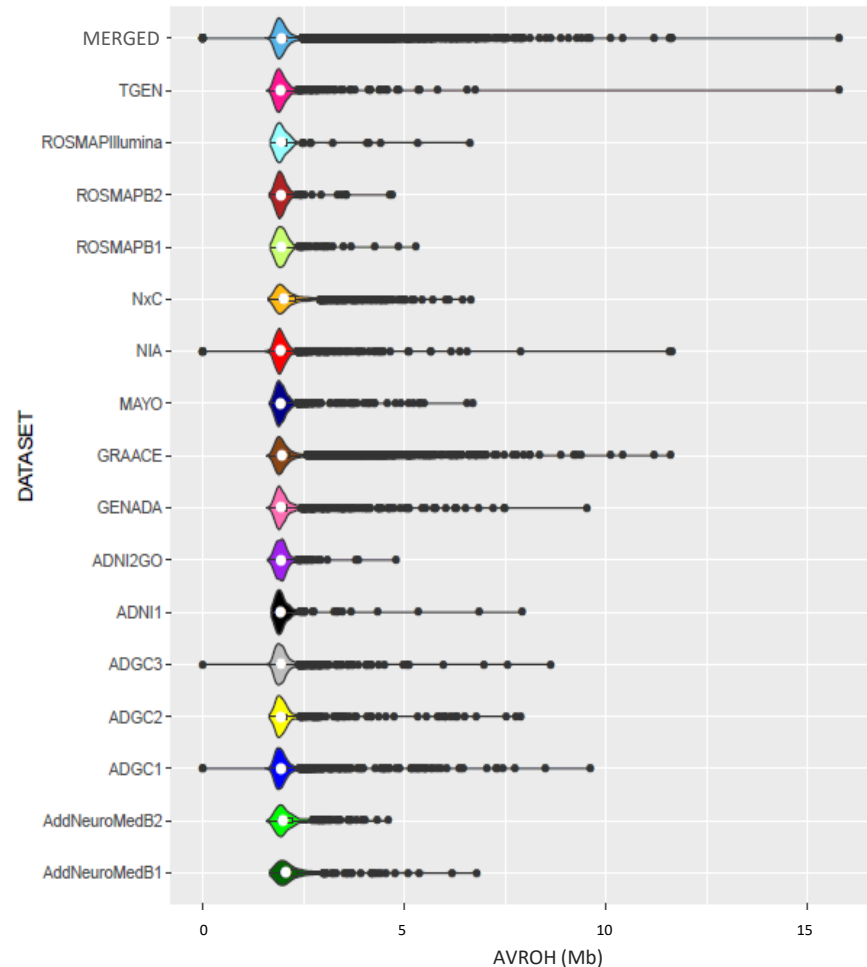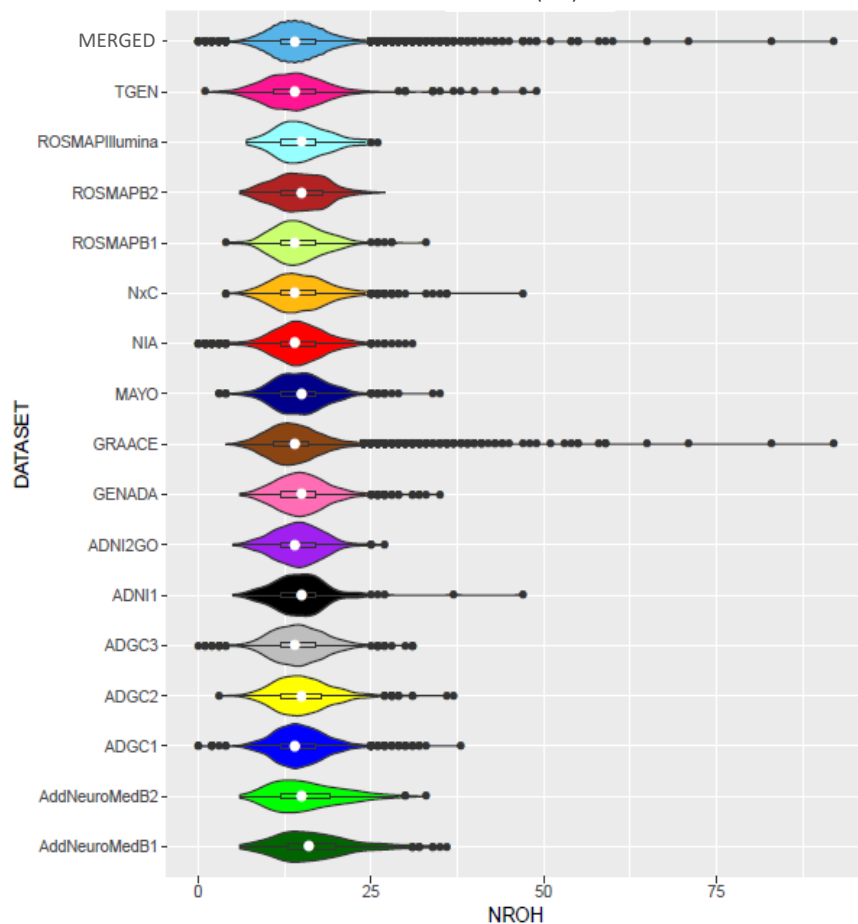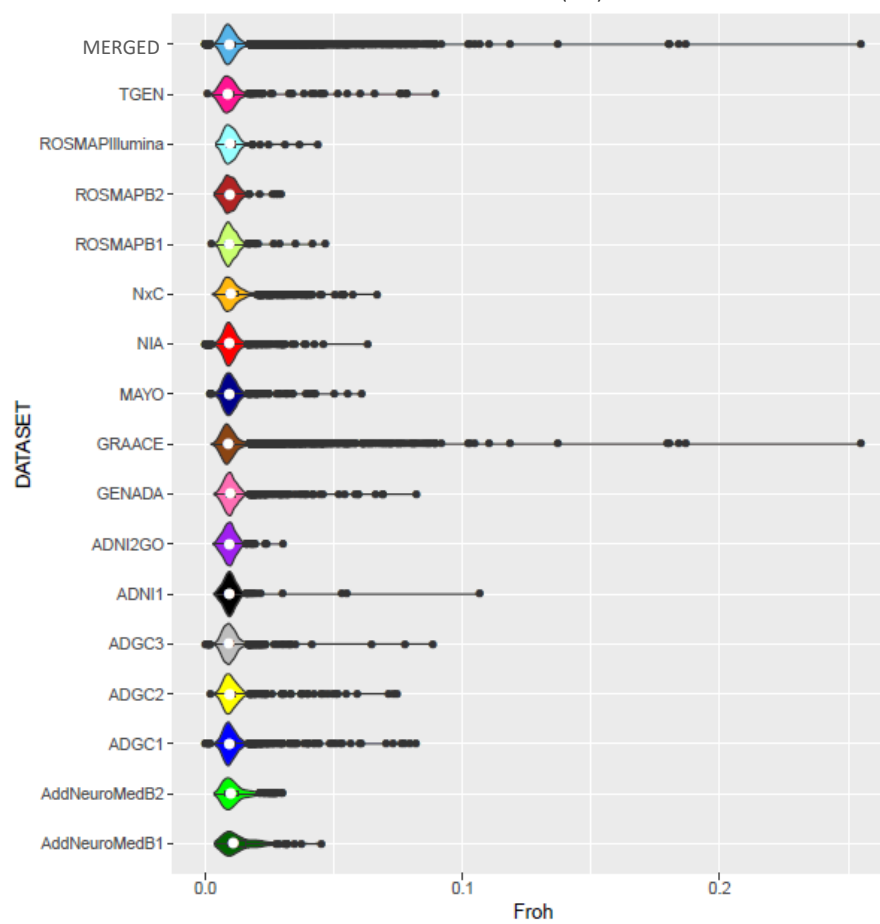

Supplement: Supplementary file 5 — Supplementary Figure 4 [file 41398_2020_1145_MOESM5_ESM.pdf]

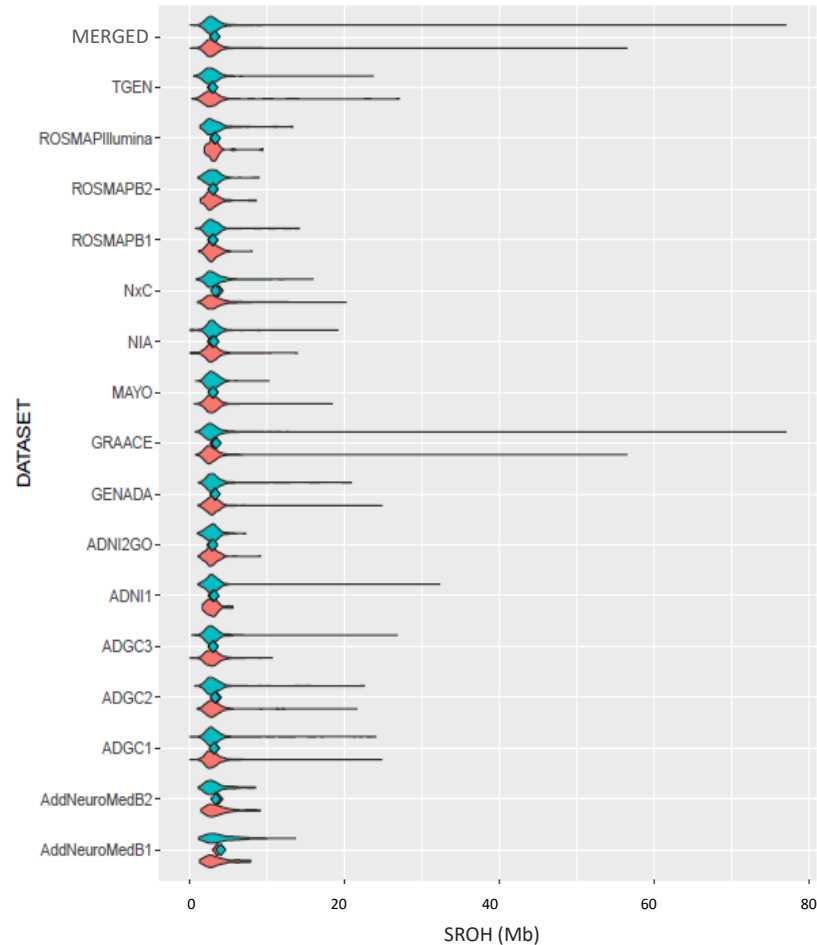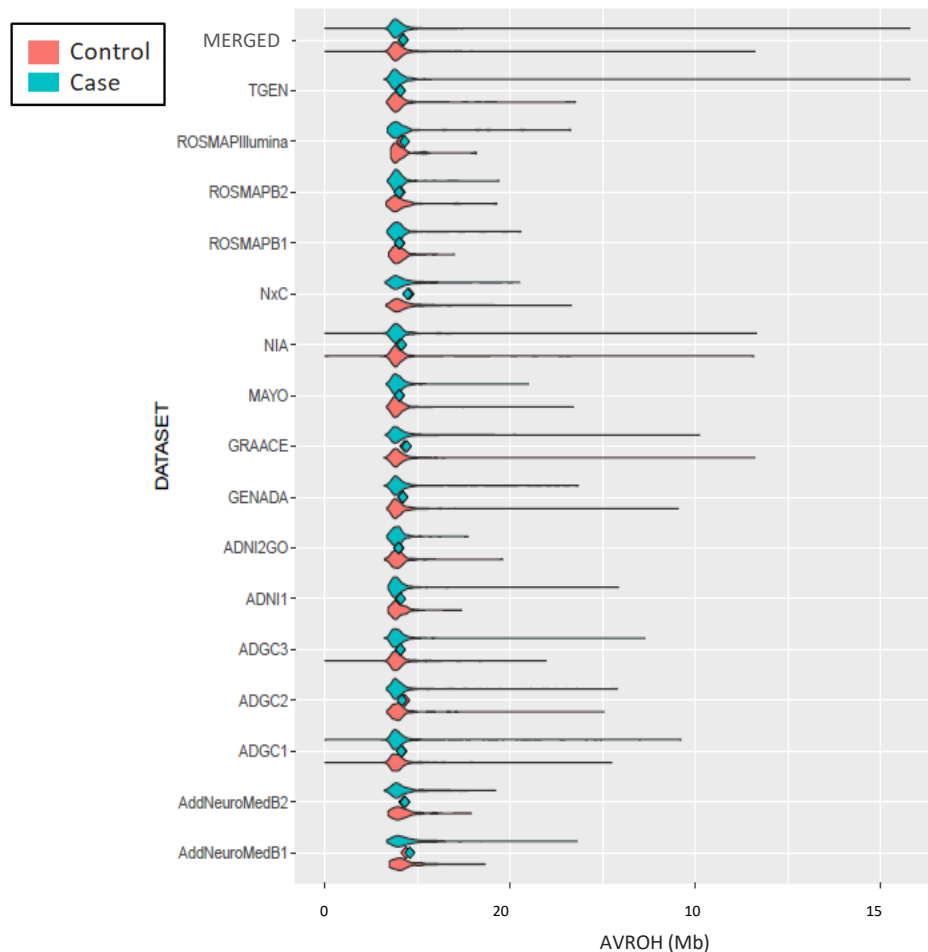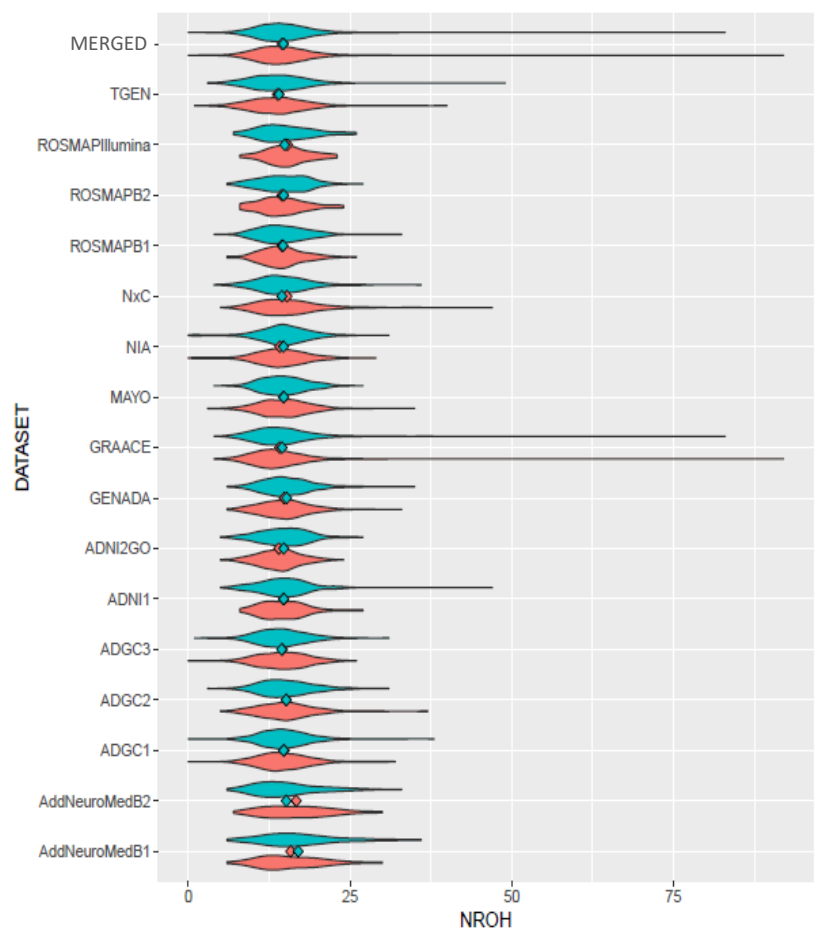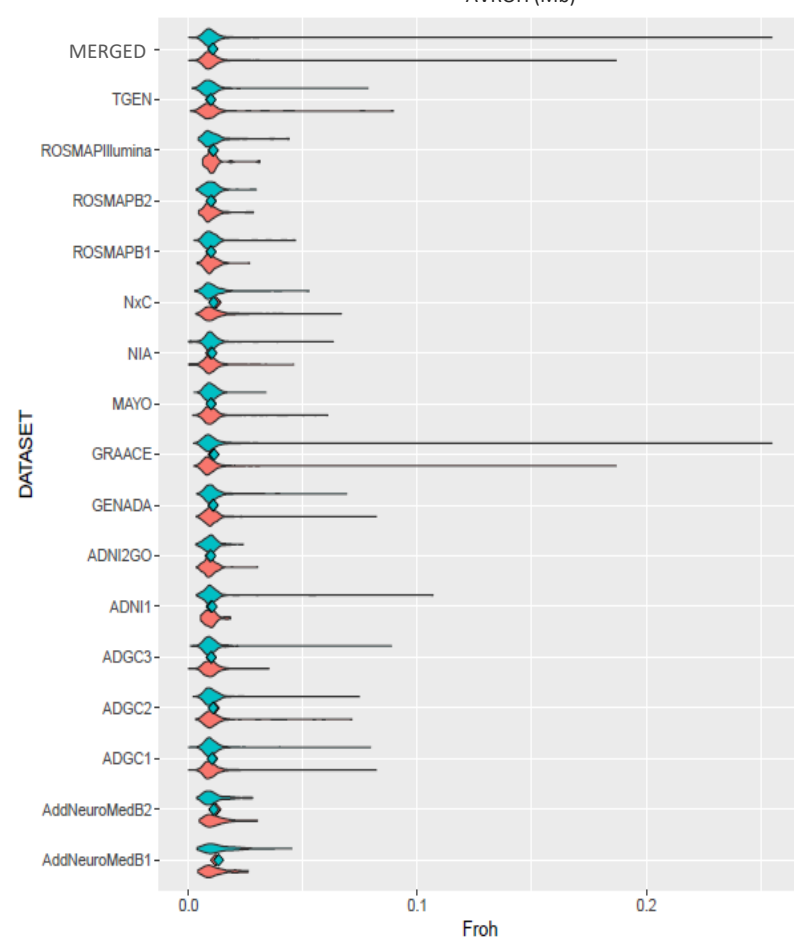

Supplement: Supplementary file 6 — Supplementary Figure 5 [file 41398_2020_1145_MOESM6_ESM.pdf]

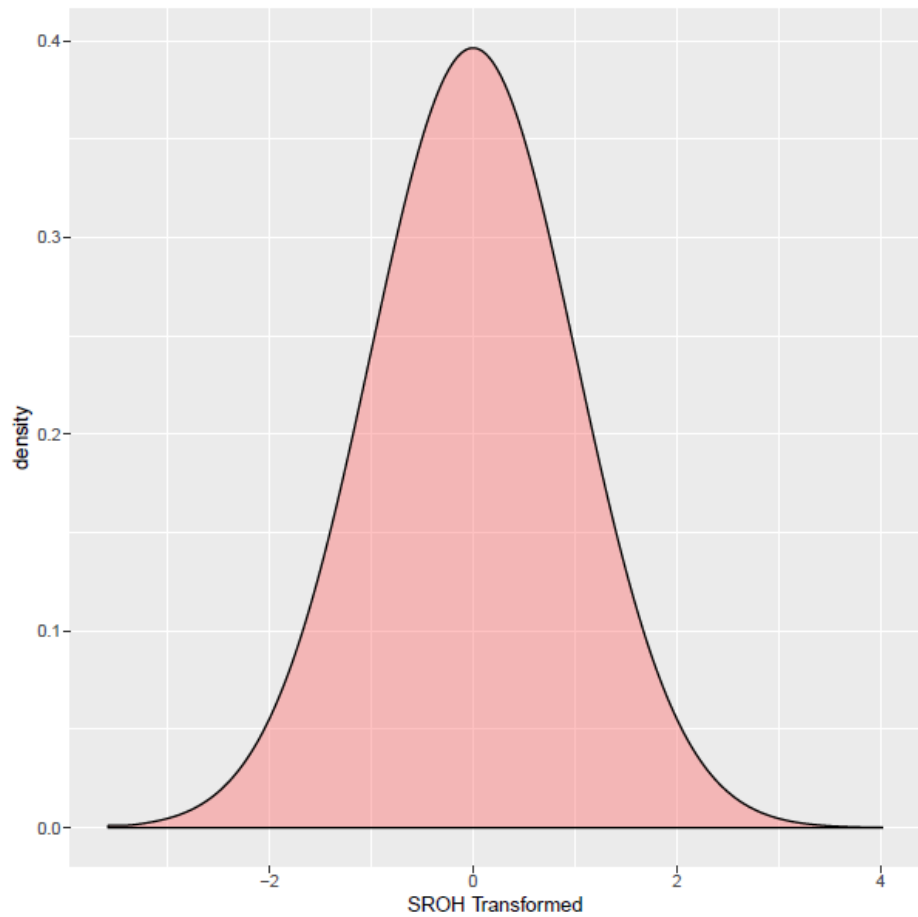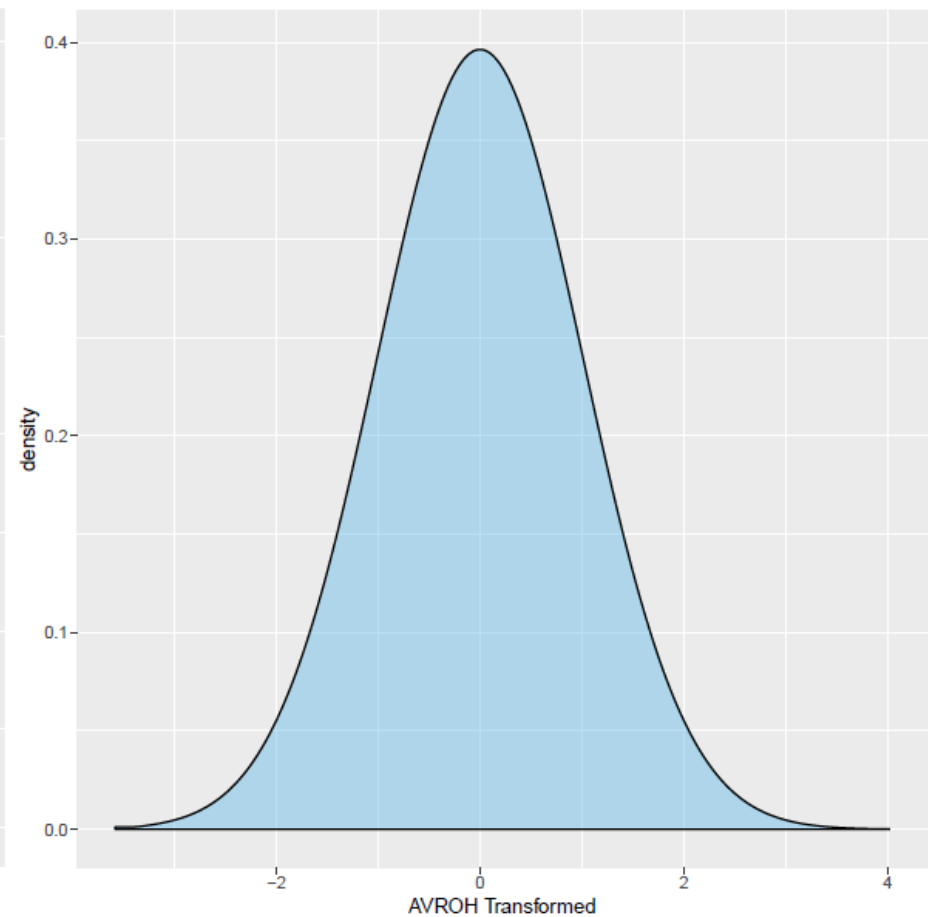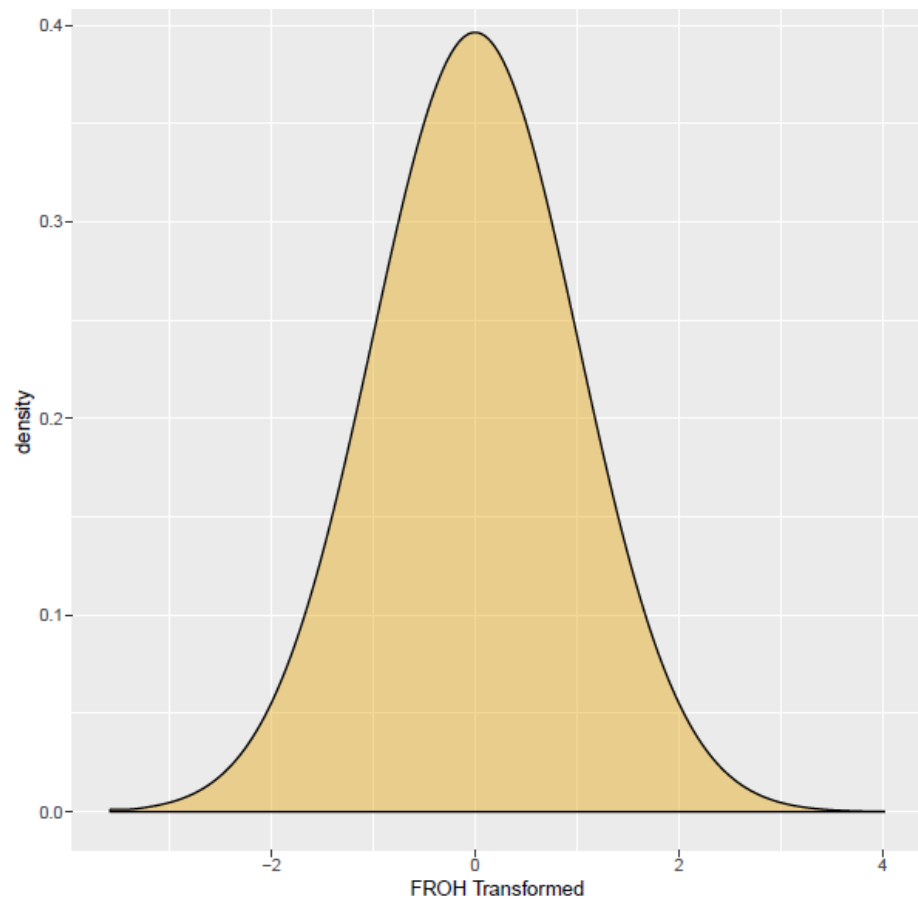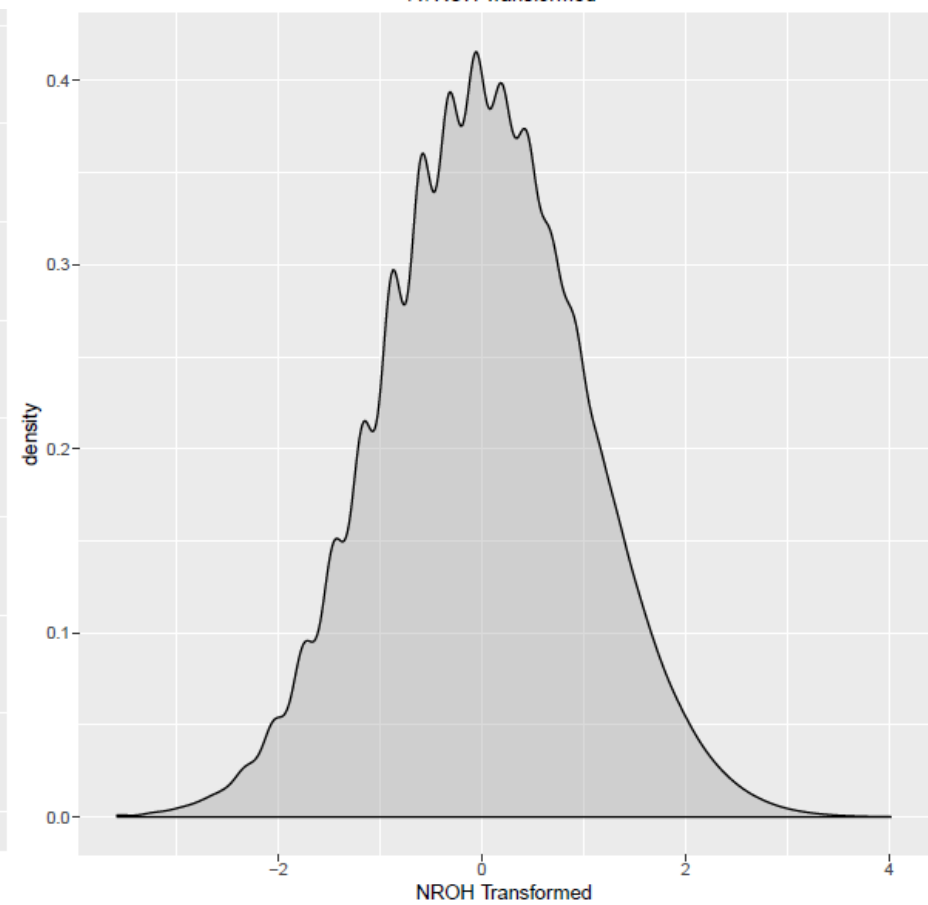

Supplement: Supplementary file 7 — Supplementary Figure 6 [file 41398_2020_1145_MOESM7_ESM.pdf]

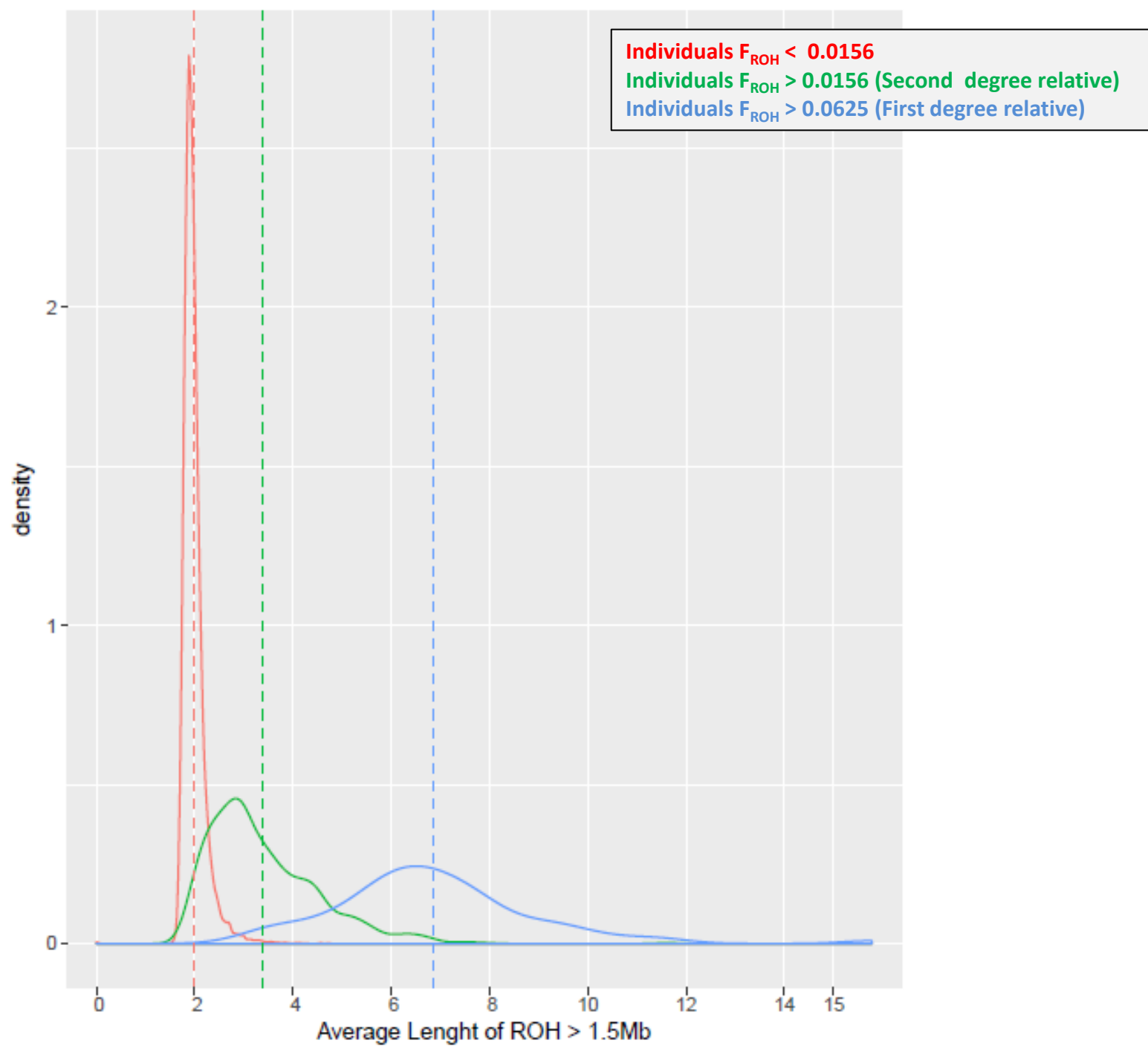

Supplement: Supplementary file 8 — Supplementary Figure 7 [file 41398_2020_1145_MOESM8_ESM.pdf]
